# Supplementary material for: Stc1: A Critical Link between RNAi and Chromatin Modification Required for Heterochromatin Integrity
Source: Cell. 2010 Mar 5;140(5):666–77. doi: 10.1016/j.cell.2010.01.038 (PMC2875855; doi:10.1016/j.cell.2010.01.038)
Supplement: Document S1. Tables S1 and S2 [file mmc1.pdf]

Table S1. List of yeast strains.

Strain	Genotype	Figure
FY1180	*h+ ade6-210 leu1-32 ura4-D18 otr1R(Sph1):ade6*$^{+}$	1, S1
FY8898	*hA clr4Δ::KanR ade6-210 leu1-32 lys1::NatR otr1R(Sph1):ade6*$^{+}$	1, S1
FY8896	*hA swi6Δ::KanR ade6-210 leu1-32 lys1::NatR otr1R(Sph1):ade6*$^{+}$	1
FY8586	*dcr1Δ::NatR ade6-210 ura4-D18/DSE otr1R(Sph1):ade6*$^{+}$	1
FY13480	*hA stc1Δ::KanR ade6-210 leu1-32 lys1::NatR otr1R(Sph1):ade6*$^{+}$	1, S1
FY4841	*h- ura4DSE leu1-32 ade6-210 his3-D1 arg3-D4 otr1R(Sph1):ura4*$^{+}$	1
FY707	*hA clr4-s5 ura4-DS/E leu1-32 ade6-210 otr1R(Sph1):ura4*$^{+}$	1
FY8538	*h+ dcr1Δ::KanR ura4-DS/E leu1-32 ade6-210 his3-D1 otr1R(Sph1):ura4*$^{+}$	1
FY13206	*h+ stc1Δ::KanR ura4-DS/E leu1-32 ade6-210 otr1R(Sph1):ura4*$^{+}$	1
FY511	*h90 mat3-M:ura4*$^{+}$ *ade6-210 leu1-32 ura4-D18*	2
FY9299	*h90 mat3-M:ura4*$^{+}$ *dcr1Δ::NatR ade6-210 leu1-32 ura4-D18*	2
FY13208	*h90 mat3-M:ura4*$^{+}$ *stc1Δ::KanR ade6-210 leu1-32 ura4-D18*	2
FY7429	*h90 mat3-M:ura4*$^{+}$ *clr4Δ:leu2*$^{+}$ *ade6-210 leu1-32 ura4-D18*	2
FY14932	*h90 dos1::kanR mat3-M:ura4*$^{+}$ *ade6-210 leu1-32 ura4-D18*	2
FY14933	*h90 dos2::kanR mat3-M:ura4*$^{+}$ *ade6-210 leu1-32 ura4-D18*	2
FY14934	*h90 rik1::kanR mat3-M:ura4*$^{+}$ *ade6-210 leu1-32 ura4-D18*	2
FY14937	*h90 mat3-M:ura4*$^{+}$ *dcr1Δ::NatR ade6-210 leu1-32 ura4-D18 [establishment]*	2
FY14938	*h90 mat3-M:ura4*$^{+}$ *stc1Δ::KanR ade6-210 leu1-32 ura4-D18 [establishment]*	2
FY8676	*h- ura4:3xgbs-ade6*$^{+}$ *ade6-DNN*	2
FY10084	*GBD-clr4-Δcd-HygR ura4:3xgbs-ade6*$^{+}$ *ade6-DNN*	2
FY14941	*dcr1Δ::NatR GBD-clr4-Δcd-HygR ura4:3xgbs-ade6*$^{+}$ *ade6-DNN [maintenance]*	2
FY14942	*rik1Δ::KanR GBD-clr4-Δcd-HygR ura4:3xgbs-ade6*$^{+}$ *ade6-DNN [maintenance]*	2
FY14943	*dos1Δ::KanR GBD-clr4-Δcd-HygR ura4:3xgbs-ade6*$^{+}$ *ade6-DNN [maintenance]*	2
FY14945	*dos2Δ::KanR GBD-clr4-Δcd-HygR ura4:3xgbs-ade6*$^{+}$ *ade6-DNN [maintenance]*	2
FY13982	*stc1Δ::KanR GBD-clr4-Δcd-HygR ura4:3xgbs-ade6*$^{+}$ *ade6-DNN [maintenance]*	2
FY14951	*dcr1Δ::NatR GBD-clr4-Δcd-HygR ura4:3xgbs-ade6*$^{+}$ *ade6-DNN [establishment]*	2
FY14952	*rik1Δ::KanR GBD-clr4-Δcd-HygR ura4:3xgbs-ade6*$^{+}$ *ade6-DNN [establishment]*	2
FY14953	*dos1Δ::KanR GBD-clr4-Δcd-HygR ura4:3xgbs-ade6*$^{+}$ *ade6-DNN [establishment]*	2
FY14954	*dos2Δ::KanR GBD-clr4-Δcd-HygR ura4:3xgbs-ade6*$^{+}$ *ade6-DNN [establishment]*	2
FY14189	*stc1Δ::KanR GBD-clr4-Δcd-HygR ura4:3xgbs-ade6*$^{+}$ *ade6-DNN [establishment]*	2
FY13721	*h+ stc1-flag-NatR ade6-210 leu1-32 ura4-D18 otr1R(Sph1):ade6*$^{+}$	3, 4, 6
FY13970	*h+ dos2-HA-NatR ade6-216 leu1-32 ura4-D18 imr1R(Nco1):ura4*$^{+}$	3
FY14104	*stc1-flag-NatR dos2-HA-NatR ade6-210 leu1-32 ura4-D18 otr1R(Sph1):ade6*$^{+}$	3,6
FY8120	*h90 rik1-myc-KanR ade6-216 leu1-32 ura4-DSE Kint2::ura4*$^{+}$	3
FY13825	*stc1-flag-NatR rik1-myc-KanR ade6-210 leu1-32 ura4-D18 otr1R(Sph1):ade6*$^{+}$	3,6
FY8635	*h+ myc-clr4 ade6-210 leu1-32 ura4-D18 otr1R(Sph1):ade6*$^{+}$	3
FY14955	*h*$^{+}$ *stc1-flag-HygR myc-clr4 ade6-210 leu1-32 ura4-D18/DSE*	3,6

FY8216	*h+ myc-ago1 ade6-704 leu1-32 ura4-D18*	3
FY13844	*stc1-flag-NatR myc-ago1 ade6-210 leu1-32 ura4-D18 otr1R(Sph1):ade6*$^+$	3,6
FY14402	*stc1-flag-NatR myc-ago1 clr4Δ::KanR ade6-210 leu1-32 ura4-D18*	3
FY14404	*stc1-flag-NatR myc-ago1 dcr1Δ::KanR ade6-210 leu1-32 ura4-D18*	3
FY14406	*stc1-flag-NatR myc-ago1 rik1Δ::KanR ade6-210 leu1-32 ura4-D18*	3
FY14408	*stc1-flag-NatR myc-ago1 dos1Δ::KanR ade6-210 leu1-32 ura4-D18*	3
FY15017	*stc1-flag-HygR myc-clr4 dos1Δ::KanR ade6-210 leu1-32 ura4-D18/DSE*	3
FY15019	*stc1-flag-HygR myc-clr4 dos2Δ::KanR ade6-210 leu1-32 ura4-D18/DSE*	3
FY15021	*stc1-flag-HygR myc-clr4 rik1Δ::KanR ade6-210 leu1-32 ura4-D18/DSE*	3
FY15023	*stc1-flag-HygR myc-clr4 dcr1Δ::NatR ade6-210 leu1-32 ura4-D18/DSE*	3
FY15357	*stc1-flag-HygR myc-clr4 ago1Δ::KanR ade6-210 leu1-32*	3
FY14598	*stc1-flag-HygR rik1-myc-KanR ade6-210 leu1-32 ura4-D18/DSE*	3
FY14600	*h*$^+$ *stc1-flag-HygR rik1-myc-KanR dcr1Δ::NatR ade6-210 leu1-32 ura4-D18/DSE*	3
FY14603	*h*$^+$ *stc1-flag-HygR rik1-myc-KanR clr4Δ::NatR ade6-210 leu1-32 ura4-D18/DSE*	3
FY14604	*h*$^+$ *stc1-flag-HygR rik1-myc-KanR dos1Δ::NatR ade6-210 leu1-32 ura4-D18/DSE*	3
FY14606	*h*$^+$ *stc1-flag-HygR rik1-myc-KanR dos2Δ::NatR ade6-210 leu1-32 ura4-D18/DSE*	3
FY15359	*h*$^+$ *stc1-flag-HygR rik1-myc-KanR ago1Δ::KanR ade6-210 leu1-32*	3
FY14410	*stc1-flag-NatR dos2-HA-NatR clr4Δ::KanR ade6-210 leu1-32 ura4-D18*	3
FY14412	*stc1-flag-NatR dos2-HA-NatR dcr1Δ::KanR ade6-210 leu1-32 ura4-D18*	3
FY14414	*stc1-flag-NatR dos2-HA-NatR rik1Δ::KanR ade6-210 leu1-32 ura4-D18*	3
FY14416	*stc1-flag-NatR dos2-HA-NatR dos1Δ::KanR ade6-210 leu1-32 ura4-D18*	3
FY15361	*stc1-flag-NatR dos2-HA-NatR ago1Δ::KanR ade6-210 leu1-32*	3
FY15669	h90 *flag-clr4 myc-ago1 ade6-210 leu1-32 ura4-D18/DSE*	3
FY15670	h90 *flag-clr4 myc-ago1 stc1Δ::NatR ade6-210 leu1-32 ura4-D18/DSE*	3
FY15671	h90 *flag-clr4 myc-ago1 dcr1Δ::NatR ade6-210 leu1-32 ura4-D18/DSE*	3
FY14177	*h+ stc1-GFP-NatR ade6-210 leu1-32 ura4-D18*	4
FY14380	*stc1-GFP-NatR clr4Δ::KanR ade6-210 leu1-32 ura4-D18/DSE*	4
FY14381	*stc1-GFP-NatR dcr1Δ::KanR ade6-210 leu1-32 ura4-D18/DSE*	4
FY13562	*h+ stc1-flag-NatR clr4Δ::KanR ade6-210 leu1-32 ura4-D18 otr1R(Sph1):ade6*$^+$	4
FY13563	*h+ stc1-flag-NatR dcr1Δ::KanR ade6-210 leu1-32 ura4-D18 otr1R(Sph1):ade6*$^+$	4
FY15005	*h+ stc1-flag-NatR ago1Δ::KanR ade6-210 leu1-32 ura4-D18 otr1R(Sph1):ade6*$^+$	
FY14106	*stc1-flag-NatR clr3Δ::KanR ade6-210 leu1-32 ura4-D18 otr1R(Sph1):ade6*$^+$	4
FY14108	*stc1-flag-NatR clr3Δ::KanR dcr1Δ::NatR ade6-210 leu1-32 ura4-D18 otr1R(Sph1):ade6*$^+$	4
FY9932	*h- flag-ago1--KanR ade6-210 leu1-32 ura4-D18*	4
FY9733	*h90 rik1-flag-NatR ade6-210 leu1-32 ura4-D18 otr1R(Sph1):ade6*$^+$	4, S4
FY8677	*h+ ura4:4xTetO-ade6*$^+$ *ade6-DNN leu1-32*	5,6
FY14394	*h+ TetR*^{off}*-flag-stc1-leu1*$^+$ *ura4:4xTetO-ade6*$^+$ *ade6-DNN leu1-32*	5,6
FY15143	*TetR*^{off}*-flag-stc1-leu1*$^+$ *ura4:4xTetO-ade6*$^+$ *dcr1Δ::NatR ade6-DNN leu1-32*	5

FY15144	*TetR^{off}-flag-stc1-leu1$^+$ ura4:4xTetO-ade6$^+$ ago1Δ::KanR ade6-DNN leu1-32*	5
FY15145	*TetR^{off}-flag-stc1-leu1$^+$ ura4:4xTetO-ade6$^+$ chp1Δ::KanR ade6-DNN leu1-32*	5
FY15146	*TetR^{off}-flag-stc1-leu1$^+$ ura4:4xTetO-ade6$^+$ cid12Δ::NatR ade6-DNN leu1-32*	5
FY15147	*TetR^{off}-flag-stc1-leu1$^+$ ura4:4xTetO-ade6$^+$ rdp1Δ::NatR ade6-DNN leu1-32*	5
FY15148	*TetR^{off}-flag-stc1-leu1$^+$ ura4:4xTetO-ade6$^+$ clr4Δ::NatR ade6-DNN leu1-32*	5
FY15149	*TetR^{off}-flag-stc1-leu1$^+$ ura4:4xTetO-ade6$^+$ sir2Δ::NatR ade6-DNN leu1-32*	5
FY15150	*TetR^{off}-flag-stc1-leu1$^+$ ura4:4xTetO-ade6$^+$ rik1Δ::KanR ade6-DNN leu1-32*	5
FY15151	*TetR^{off}-flag-stc1-leu1$^+$ ura4:4xTetO-ade6$^+$ dos1Δ::KanR ade6-DNN leu1-32*	5
FY15152	*TetR^{off}-flag-stc1-leu1$^+$ ura4:4xTetO-ade6$^+$ dos2Δ::KanR ade6-DNN leu1-32*	5
FY15012	*h+ stc1-K100A-flag-NatR ade6-210 leu1-32 ura4-D18 otr1R(Sph1):ade6$^+$*	6, S5
FY15014	*h+ stc1-R116A-flag-NatR ade6-210 leu1-32 ura4-D18 otr1R(Sph1):ade6$^+$*	6, S5
FY15366	*stc1-K100A-flag-NatR dos2-HA-NatR ade6-210 leu1-32 ura4-D18*	6
FY15368	*stc1-R116A-flag-NatR dos2-HA-NatR ade6-210 leu1-32 ura4-D18*	6
FY15140	*stc1-K100A-flag-NatR rik1-myc-KanR ade6-210 leu1-32 ura4-D18*	6
FY15142	*stc1-R116A-flag-NatR rik1-myc-KanR ade6-210 leu1-32 ura4-D18*	6
FY15162	*stc1-K100A-flag-NatR myc-clr4 ade6-210 leu1-32 ura4-D18/DSE*	6
FY15164	*stc1-R116A-flag-NatR myc-clr4 ade6-210 leu1-32 ura4-D18/DSE*	6
FY15225	*stc1-K100A-flag-NatR myc-ago1 ade6-210 leu1-32 ura4-D18 otr1R(Sph1):ade6$^+$*	6
FY15227	*stc1-R116A-flag-NatR myc-ago1 ade6-210 leu1-32 ura4-D18 otr1R(Sph1):ade6$^+$*	6
FY15513	*h+ TetR^{off}-flag-stc1-K100A-leu1$^+$ ura4:4xTetO-ade6$^+$ ade6-DNN leu1-32*	6
FY15515	*h+ TetR^{off}-flag-stc1-R116A-leu1$^+$ ura4:4xTetO-ade6$^+$ ade6-DNN leu1-32*	6
FY1863	*tel1:his3 ade6-210 leu1-32 ura4-D18 his3-D1 otr1R(Sph1):ade6$^+$*	S2
FY1966	*clr4-S5 tel1:his3 ade6-210 leu1-32 ura4-D18 his3-D1 otr1R(Sph1):ade6$^+$*	S2
FY2506	*rik1Δ::leu2+ tel1:his3 ade6-210 leu1-32 ura4-DSE his3-D1 otr1R(Sph1):ade6$^+$*	S2
FY15382	*dcr1Δ::NatR tel1:his3 ade6-210 leu1-32 ura4-D18/DSE his3-D1*	S2
FY15384	*stc1Δ::NatR tel1:his3 ade6-210 leu1-32 ura4-D18/DSE his3-D1*	S2
FY15386	*dos1Δ::NatR tel1:his3 ade6-210 leu1-32 ura4-D18/DSE his3-D1*	S2
FY15388	*dos2Δ::NatR tel1:his3 ade6-210 leu1-32 ura4-D18/DSE his3-D1*	S2
FY14359	*h- GFP-dos1-ura4$^+$ leu1-32 ura4-D18*	S3
FY14545	*stc1-flag-NatR GFP-dos1-ura4$^+$ ade6-210 leu1-32 ura4-D18 otr1R(Sph1):ade6$^+$*	S3
FY8923	*h+ cid12-HA-KanR ade6-210 leu1-32 ura4-DSE arg3D4 his3D1*	S3
FY13827	*stc1-flag-NatR cid12-HA-KanR ade6-210 leu1-32 ura4-D18 otr1R(Sph1):ade6$^+$*	S3
FY12406	*h+ rdp1-HA-KanR ade6-210 leu1-32 ura4-D18 otr1R(Sph1):ade6$^+$*	S3
FY13829	*stc1-flag-NatR rdp1-HA-KanR ade6-210 leu1-32 ura4-D18 otr1R(Sph1):ade6$^+$*	S3
FY12191	*h+ rdp1-myc-hygR ade6-210 leu1-32 ura4-DSE/D18*	S3
FY15351	*chp1-flag-KanR rdp1-myc-hygR ade6-210/216 leu1-32 ura4-DSE/D18*	S3
FY15459	*chp1-flag-KanR rdp1-myc-hygR stc1Δ::NatR ade6-210/216 leu1-32 ura4-DSE*	S3
FY15461	*chp1-flag-KanR rdp1-myc-hygR dcr1Δ::NatR ade6-210/216 leu1-32 ura4-DSE*	S3
FY14669	*rik1-flag-NatR GFP-dos1-ura4$^+$ ade6-210 leu1-32 ura4-D18 otr1R(Sph1):ade6$^+$*	S3

FY14844	rik1-flag-NatR GFP-dos1-ura4^{+} stc1Δ::KanR ade6-210 leu1-32 ura4-D18 otr1R(Sph1):ade6^{+}	S3
FY14678	dos2-flag-NatR GFP-dos1-ura4^{+} ade6-210 leu1-32 ura4-D18 otr1R(Sph1):ade6^{+}	S3
FY14852	dos2-flag-NatR GFP-dos1-ura4^{+} stc1Δ::KanR ade6-210 leu1-32 ura4-D18 otr1R(Sph1):ade6^{+}	S3
FY14665	h90 stc1-flag-HygR mat3-M::ura4+ leu1-32 ura4-D18	S4
FY14758	h90 stc1-flag-HygR dcr1Δ::NatR mat3-M::ura4+ leu1-32 ura4-D18	S4
FY15363	h90 stc1-flag-HygR clr4Δ::leu2+ mat3-M::ura4+ leu1-32 ura4-D18	S4
FY8119	h90 flag-clr4 ade6-210 leu1-32 ura4-DSE Kint2::ura4	S4
FY14111	h+ dos2-flag-NatR ade6-210 leu1-32 ura4-D18 otr1R(Sph1):ade6^{+}	S4
FY15624	stc1-flag-HygR rik1-flag-NatR ade6-210 leu1-32 ura4-D18 otr1R(Sph1):ade6^{+}	S4
FY15392	stc1-flag-NatR GBD-clr4-Δcd-HygR ura4:3xgbs-ade6^{+} ade6-DNN [establishment]	S5
FY15395	stc1-K100A-flag-NatR GBD-clr4-Δcd-HygR ura4:3xgbs-ade6^{+} ade6-DNN [establishment]	S5
FY15398	stc1-R116A-flag-NatR GBD-clr4-Δcd-HygR ura4:3xgbs-ade6^{+} ade6-DNN [establishment]	S5

Table S2. List of primers used in this study.

**qPCR**	
q_cen(dg)_FOR	AATTGTGGTGGTGTGGTAATAC
q_cen(dg)_REV	GGGTTCATCGTTTCCATTCAG
q_ade6_FOR	ATGCTTATCCTACAACTGAGACC
q_ade6_REV	TGAATTGAGAAGGGAAGACGAG
q_ura4_FOR	CGTGGTCTCTTGCT TTGG
q_ura4_REV	GTAGTCGCTTTGAAGGTTAGG
q_act_FOR	GGTTTCGCTGGAGATGATG
q_act_REV	ATACCACGCTTGCTTTGAG
q_ura4-5'_FOR	TCGCAGACATTGGAAATACC
q_ura4-5'_REV	ATGGCAATTTGTGATATGAGC
q_tlh_FOR	GGATAAGCCAATCATCGTTGAG
q_tlh_REV	GTAGTTGACGCTCCTTGGAAG
q_mat_FOR	GTCCGAGGCAATACAACTTTGG
q_mat_REV	GGTTGACAGTAGGAGATATTTACAG
**Northern probes**	
censiRNAs	
IK8	ATTCCTTTCTGAACCTCTCTGTTAT
IK9	TTTGATGCCCATGTTCATTCCACTTG
IK10	GGGAGTACATCATTCCTACTTCGATA
snR58	GATGAAATTCAGAAGTCTAGCATC
