# Supplementary material for: Stc1: A Critical Link between RNAi and Chromatin Modification Required for Heterochromatin Integrity
Source: Cell. 2010 Mar 5;140(5):666–77. doi: 10.1016/j.cell.2010.01.038 (PMC2875855; doi:10.1016/j.cell.2010.01.038)
Supplement: Document S3. Article Plus Supplemental Information [file mmc3.pdf]

Cell

Stc1: A Critical Link between RNAi and Chromatin Modification Required for Heterochromatin Integrity

Elizabeth H. Bayne,[1] Sharon A. White,[1] Alexander Kagansky,[1] Dominika A. Bijos,[1,5] Luis Sanchez-Pulido,[2] Kwang-Lae Hoe,[3] Dong-Uk Kim,[3] Han-Oh Park,[4] Chris P. Ponting,[2] Juri Rappsilber,[1] and Robin C. Allshire[1,*]
[1]Wellcome Trust Centre for Cell Biology and Institute of Cell Biology, School of Biological Sciences, The University of Edinburgh, Edinburgh EH9 3JR, Scotland, UK
[2]MRC Functional Genomics Unit, University of Oxford, South Parks Road, Oxford OX1 3QX, UK
[3]Integrative Omics Research Center, Korea Research Institute of Bioscience and Biotechnology, Daejeon 305-806, Republic of Korea
[4]Bioneer Corporation, Daejeon 306-220, Republic of Korea
[5]Present address: Division of Gene Regulation, Netherlands Cancer Institute, Plesmanlaan 121, 1066 CX Amsterdam, The Netherlands
*Correspondence: robin.allshire@ed.ac.uk
DOI 10.1016/j.cell.2010.01.038

SUMMARY

In fission yeast, RNAi directs heterochromatin formation at centromeres, telomeres, and the mating type locus. Noncoding RNAs transcribed from repeat elements generate siRNAs that are incorporated into the Argonaute-containing RITS complex and direct it to nascent homologous transcripts. This leads to recruitment of the CLRC complex, including the histone methyltransferase Clr4, promoting H3K9 methylation and heterochromatin formation. A key question is what mediates the recruitment of Clr4/CLRC to transcript-bound RITS. We have identified a LIM domain protein, Stc1, that is required for centromeric heterochromatin integrity. Our analyses show that Stc1 is specifically required to establish H3K9 methylation via RNAi, and interacts both with the RNAi effector Ago1, and with the chromatin-modifying CLRC complex. Moreover, tethering Stc1 to a euchromatic locus is sufficient to induce silencing and heterochromatin formation independently of RNAi. We conclude that Stc1 associates with RITS on centromeric transcripts and recruits CLRC, thereby coupling RNAi to chromatin modification.

INTRODUCTION

RNA interference (RNAi) is a silencing mechanism widely employed in eukaryotes. Divergent RNAi pathways mediate silencing in a variety of ways but all are characterized by small RNAs that are bound by Argonaute effector proteins and act as specificity factors to target homologous sequences for repression (Ghildiyal and Zamore, 2009). RNAi frequently acts at the posttranscriptional level, reducing gene expression by directing transcript cleavage or translational inhibition. However, RNAi can also trigger DNA and/or chromatin modifications that lead to transcriptional silencing and heterochromatin formation. Such RNA-directed chromatin modification is best understood in the fission yeast *Schizosaccharomyces pombe* (Bernstein and Allis, 2005; Bühler and Moazed, 2007).

In fission yeast, domains of heterochromatin are found at telomeres, the silent mating-type locus, and on pericentromeric repeats (reviewed in Bühler and Moazed, 2007; Grewal and Jia, 2007). This heterochromatin is characterized by histone H3 lysine 9 methylation (H3K9me), mediated by the sole H3K9 methyltransferase, Clr4. H3K9me creates binding sites for the chromodomain proteins Swi6, Chp1, Chp2, and Clr4 (Bannister et al., 2001; Sadaie et al., 2004; Zhang et al., 2008). Several histone deacetylases (HDACs), Clr3, Clr6, and Sir2, are also required to facilitate H3K9 methylation (Grewal et al., 1998; Nakayama et al., 2001; Shankaranarayana et al., 2003). At centromeres, RNAi promotes H3K9 methylation on centromeric outer repeat sequences (Motamedi et al., 2004; Verdel et al., 2004; Volpe et al., 2002). It is possible to distinguish between establishment of H3K9me, which is fully dependent on RNAi, and its subsequent maintenance, which is only partially RNAi dependent (Sadaie et al., 2004). RNAi also targets mating-type locus and telomeric elements with homology to centromere outer repeats. However, here, alternative pathways act redundantly with RNAi to recruit chromatin modifiers, so that RNAi is required for establishment but not for maintenance of H3K9me at these loci (Hansen et al., 2006; Jia et al., 2004; Kanoh et al., 2005; Kim et al., 2004).

RNAi in fission yeast is triggered by double-stranded RNA (dsRNA) derived from noncoding centromere outer repeat transcripts produced during S phase by RNA polymerase II (reviewed in Bühler and Moazed, 2007; Grewal and Jia, 2007; Kloc and Martienssen, 2008). Dicer (Dcr1) cleaves these dsRNA molecules into short interfering RNAs (siRNAs) that guide the Argonaute (Ago1)-containing RITS effector complex to homologous nascent transcripts by sequence complementarity. Association of the RITS complex (Ago1, Tas3, and Chp1) with chromatin is facilitated by binding of the chromodomain protein Chp1 to H3K9me nucleosomes, which drives a self-enforcing

loop coupling spreading of H3K9me with RITS binding. Nascent transcript-bound RITS also recruits the RNA-directed RNA polymerase complex (RDRC; Rdp1, Cid12, and Hrr1), which may promote further dsRNA and siRNA production. By a mechanism that is not understood, this cotranscriptional form of RNAi can recruit Clr4 to initiate H3K9me. H3K9me then spreads to form a heterochromatin domain (reviewed in Bühler and Moazed, 2007; Grewal and Jia, 2007).

Clr4 is associated with a multisubunit complex containing Rik1, Dos1 (Raf1/Cmc1/Clr8), Dos2 (Raf2/Cmc2/Clr7), and Pcu4/Cul4 (Hong et al., 2005; Horn et al., 2005; Jia et al., 2005; Li et al., 2005; Thon et al., 2005). This Clr4-Rik1-Cul4 complex (CLRC) is an active Cullin-dependent E3 ubiquitin ligase essential for heterochromatin assembly. Rik1 has a WD40/β-propeller domain similar to damaged DNA-binding protein DDB1 (Neuwald and Poleksic, 2000). Dos1 also contains WD40 repeats, while Dos2 has no obvious domains. Cul4 serves as a scaffold for ubiquitin ligase assembly, and must be neddylated for cullin-dependent ubiquitin ligase activity. Although the relationship between the ubiquitin ligase activity of CLRC and Clr4-mediated heterochromatin formation is unclear, these Clr4-associated factors are all required for H3K9 methylation and heterochromatin integrity (Hong et al., 2005; Horn et al., 2005; Jia et al., 2005; Li et al., 2005; Thon et al., 2005). A critical question is what connects this Clr4 methyltransferase complex CLRC to the RNAi machinery, to mediate its RNAi-dependent recruitment to chromatin.

It has been shown previously that Rik1 and Clr4 associate with the RITS component Chp1, and that Rik1 recruitment to the centromeric repeats is enhanced when production of centromere transcripts and siRNAs is increased (Zhang et al., 2008). However, what mediates the association of CLRC with RITS is unknown. In a genome-wide screen, we identified *stc1*$^{+}$ as a gene required for heterochromatin integrity. Stc1 is a LIM domain protein that associates with CLRC, and additionally associates with the RNAi effector protein Ago1. The phenotypes of *stc1*Δ cells resemble those of RNAi mutants; however, artificially tethering Stc1 to a euchromatic locus promotes H3K9 methylation and heterochromatin formation independently of RNAi. Our analyses lead us to conclude that once RITS/Ago1 has been guided to centromeric transcripts by siRNAs, Stc1 is the key protein that recruits CLRC. Stc1 is therefore critical for the recognition of ongoing cotranscriptional RNAi and delivery of the chromatin modifiers that promote heterochromatin formation.

RESULTS

Stc1 Is Required for Heterochromatin Integrity

In a screen of ~3000 strains bearing single nonessential gene deletions (E.H.B., D.A.B., and R.C.A., unpublished data), we identified SPBP8B7.28c as a gene required for marker gene silencing in outer repeat heterochromatin of centromere 1 (*cen1:ade6*$^{+}$; Figure 1). We named this gene *stc1*$^{+}$ (siRNA *to* chromatin). Plating assays and quantitative RT-PCR (qRT-PCR) analyses confirmed that, as in cells lacking other components of the RNAi-directed chromatin modification process (*clr4*Δ, *swi6*Δ, and *dcr1*Δ), deletion of *stc1*$^{+}$ (*stc1*Δ) results in alleviation of silencing of *cen1:ade6*$^{+}$ and accumulation of noncoding outer repeat transcripts (Figures 1A and 1B). Conversely, siRNAs homologous to outer repeats are not detectable in *stc1*Δ cells (Figure 1C). siRNAs are also undetectable in cells lacking RNAi components (*dcr1*Δ, *chp1*Δ, *ago1*Δ, and *tas3*Δ); however, we find that low levels of siRNAs clearly remain in CLRC complex mutants (*clr4*Δ, *rik1*Δ, *dos1*Δ, and *dos2*Δ). The absence of detectable siRNAs in *stc1*Δ cells therefore implicates Stc1 in the RNAi pathway.

Perturbation of centromeric heterochromatin causes defects in chromosome segregation (Allshire et al., 1995; Ekwall et al., 1995; Hall et al., 2003; Volpe et al., 2003). We found that, as with other RNAi and heterochromatin mutants, *stc1*Δ cells lose a minichromosome at elevated rates, are sensitive to the microtubule destabilizing compound TBZ (Figure S1 available online), and display a high incidence of lagging chromosomes in late anaphase, indicative of defects in centromere function (Figure 1D).

Cells lacking CLRC subunits (*clr4*Δ, *rik1*Δ, *cul4*Δ, *dos1*Δ, or *dos2*Δ) exhibit delocalization of Swi6 and complete loss of H3K9me from heterochromatic regions (Ekwall et al., 1996; Hong et al., 2005; Horn et al., 2005; Jia et al., 2005; Li et al., 2005; Thon et al., 2005). In contrast, cells lacking RNAi (i.e., *dcr1*Δ) retain some H3K9me on heterochromatic repeats, albeit at reduced levels, but lose it from embedded marker genes such as *cen1:ade6*$^{+}$ or *cen1:ura4*$^{+}$ (Bayne et al., 2008; Sadaie et al., 2004; Yamada et al., 2005). Anti-H3K9me2 chromatin immunoprecipitation (ChIP) on *stc1*Δ cells indicates that, as in *dcr1*Δ cells, H3K9me2 is only partially reduced on centromeric outer repeats (*cen-dg*) but is completely lost from the *cen1:ura4*$^{+}$ marker gene. Consistent with this, a reduced level of Swi6 remains associated with outer repeats, but not with *cen1:ura4*$^{+}$ (Figure 1E). Thus, like Dcr1, Stc1 is required to process centromeric transcripts into siRNAs, but is not absolutely required to maintain H3K9me2 and Swi6 on centromere repeats. This suggests that Stc1 is required for H3K9 methylation, and thus heterochromatin formation, via RNAi.

Stc1 Is Required to Establish, but Not to Maintain, Mating-Type Locus and Tethered Clr4 Silencing

It is known that RNAi is required to establish, but not to maintain, silent heterochromatin over the *mat2-mat3* and subtelomeric regions. Consequently, deletion of Dcr1 has little or no impact on silencing of embedded genes (Hall et al., 2002; Kanoh et al., 2005). We crossed *stc1*Δ, *dcr1*Δ, *clr4*Δ, *rik1*Δ, *dos1*Δ, or *dos2*Δ cells with cells harboring a silent *ura4*$^{+}$ gene close to *mat3* (*mat3-M:ura4*$^{+}$). The CLRC components Clr4, Rik1, Dos1, and Dos2 are required to maintain silencing of *mat2-mat3*; consequently, cells lacking these proteins lose *mat3-M:ura4*$^{+}$ silencing, resulting in growth on plates lacking uracil (−URA) and loss of resistance to counterselective FOA (+FOA). qRT-PCR confirms that *ura4*$^{+}$ transcript levels increase by around 20-fold in CLRC mutants (Figures 2A–2C). In contrast, *mat3-M:ura4*$^{+}$ remained silent in both *dcr1*Δ and *stc1*Δ cells, as indicated by continued growth on FOA (equivalent to the wild-type) and low transcript levels. Maintenance of silencing at telomeres was similarly unaffected by deletion of Dcr1 or Stc1 but was disrupted by deletion of CLRC components (Figure S2).

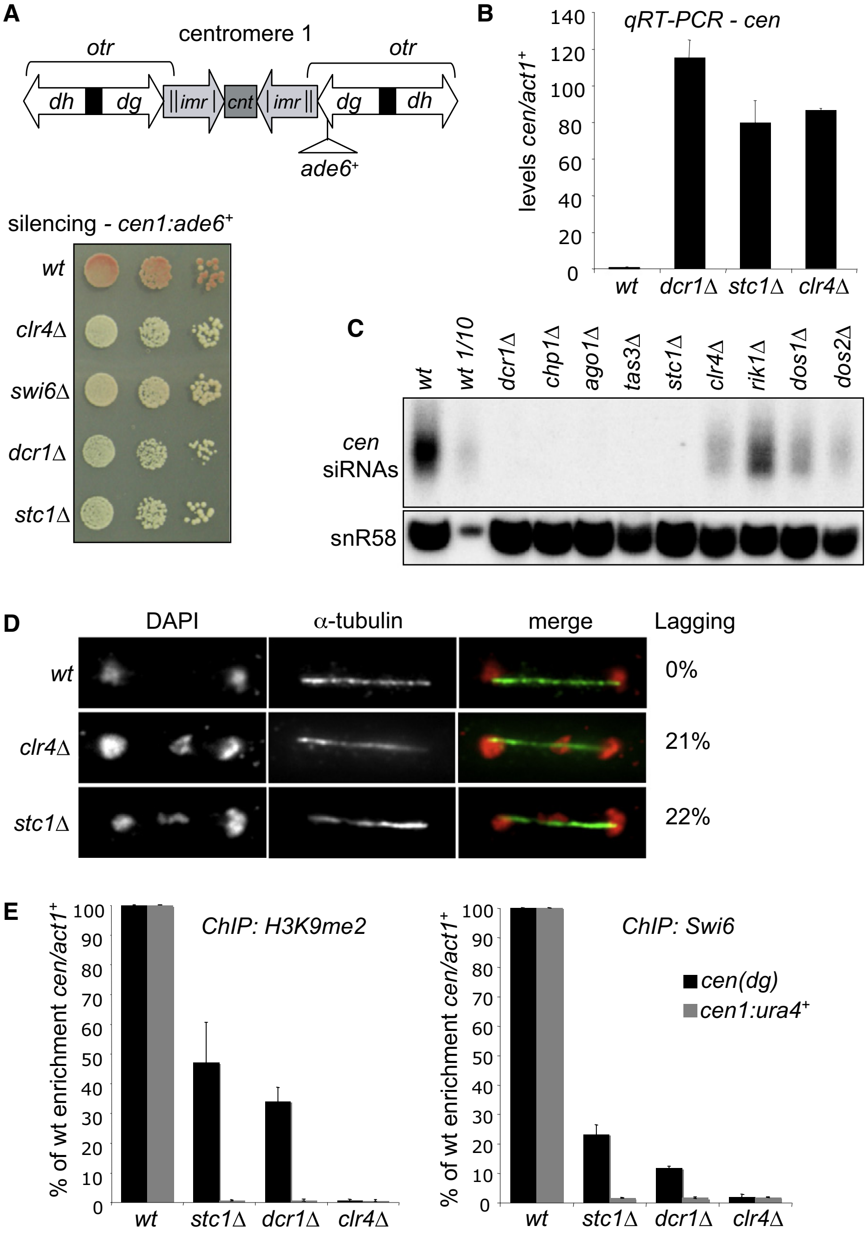

Figure 1. Stc1 Is Required for RNAi-Directed Silencing at Centromeres

(A) Assay for silencing at *cen1:ade6*+. Diagram shows position of the *cen1:ade6*+ marker gene in centromere 1, relative to outer repeat (*otr*) *dg* and *dh* elements, inner repeats (*imr*), and central core (*cnt*). Wild-type cells with silenced *cen1:ade6*+ form red colonies on limiting adenine; loss of silencing leads to pink/white colonies.

(B) qRT-PCR analysis of *cen(dg)* transcript levels relative to a control transcript *act1*+, normalized to the wild-type.

(C) Northern analysis of centromeric siRNAs, including a dilution series for wild-type. Loading control: snoRNA58 (snR58).

(D) Analysis of lagging chromosomes in anaphase by fluorescence microscopy. Representative images show fixed cells stained for DNA (DAPI—red) and tubulin (green). Percentages of anaphase cells with lagging chromosomes are indicated (n = 100).

(E) ChIP analysis of H3K9me2 and Swi6 levels associated with *cen(dg)* or *cen1:ura4*+, relative to *act1*+, and normalized to wild-type.

See also Figure S1. All error bars indicate standard deviation (SD).

So that the role of Stc1 in establishing silencing of *mat3-M:ura4*+ could be assessed, the *clr4*+ gene was removed (causing loss of silencing) and then reintroduced in wild-type, *dcr1*Δ or *stc1*Δ backgrounds by crossing (Figure 2A). Silencing of *mat3:ura4*+ could not be re-established in the absence of Dcr1 or Stc1 (Figures 2B and 2C). This indicates that, like Dcr1, Stc1 is required for the establishment, but not the maintenance, of silencing over *mat2-mat3*, strongly suggesting that Stc1 is specifically required for RNAi-dependent heterochromatin establishment.

We recently showed that artificial recruitment of Clr4 to heterologous DNA-binding sites induces heterochromatin formation and silencing of nearby marker genes (Kagansky et al., 2009). In this system, cells express Clr4 (chromodomain deleted) fused to the Gal4-binding domain (GBD-Clr4-Δcd). Recruitment of this GBD-Clr4 fusion protein to Gal4-binding sites upstream of *ade6*+ (*3xgbs-ade6*+) silences *ade6*+ expression, resulting in red colonies (as opposed to white, *ade6*+-expressing, colonies). Both establishment and maintenance of this synthetic heterochromatin are essentially RNAi independent, indicating that tethering Clr4 methyltransferase activity to DNA bypasses the need for RNAi-mediated Clr4 recruitment. In contrast, the CLRC component Rik1 is critical for both establishment and maintenance of this heterochromatin (Kagansky et al., 2009). We tested the contribution of Stc1 to this synthetic heterochromatin, comparing it to that of Dcr1, Rik1, Dos1, and Dos2 using plating assays and qRT-PCR (Figures 2D and 2E). Deletion of Stc1 had no effect on pre-established GBD-Clr4-Δcd-mediated silencing of *3xgbs-ade6*+, as indicated by red colony color and low *ade6*+ transcript levels, similar to *dcr1*Δ and wild-type cells. In contrast, the CLRC mutants, *rik1*Δ, *dos1*Δ, and *dos2*Δ, produced white colonies and high transcript levels, indicative of loss of silencing. Thus, maintenance of GBD-Clr4-Δcd-mediated silencing requires CLRC components but not Stc1 or RNAi (Dcr1), consistent with Stc1 being required for RNAi-mediated CLRC recruitment. However, when GBD-Clr4-Δcd and the *3xgbs-ade6*+ reporter were freshly combined in the absence of Stc1, *ade6*+ was not silenced, as shown by white colonies and high transcript levels (Figures 2D and 2E). This indicates that,

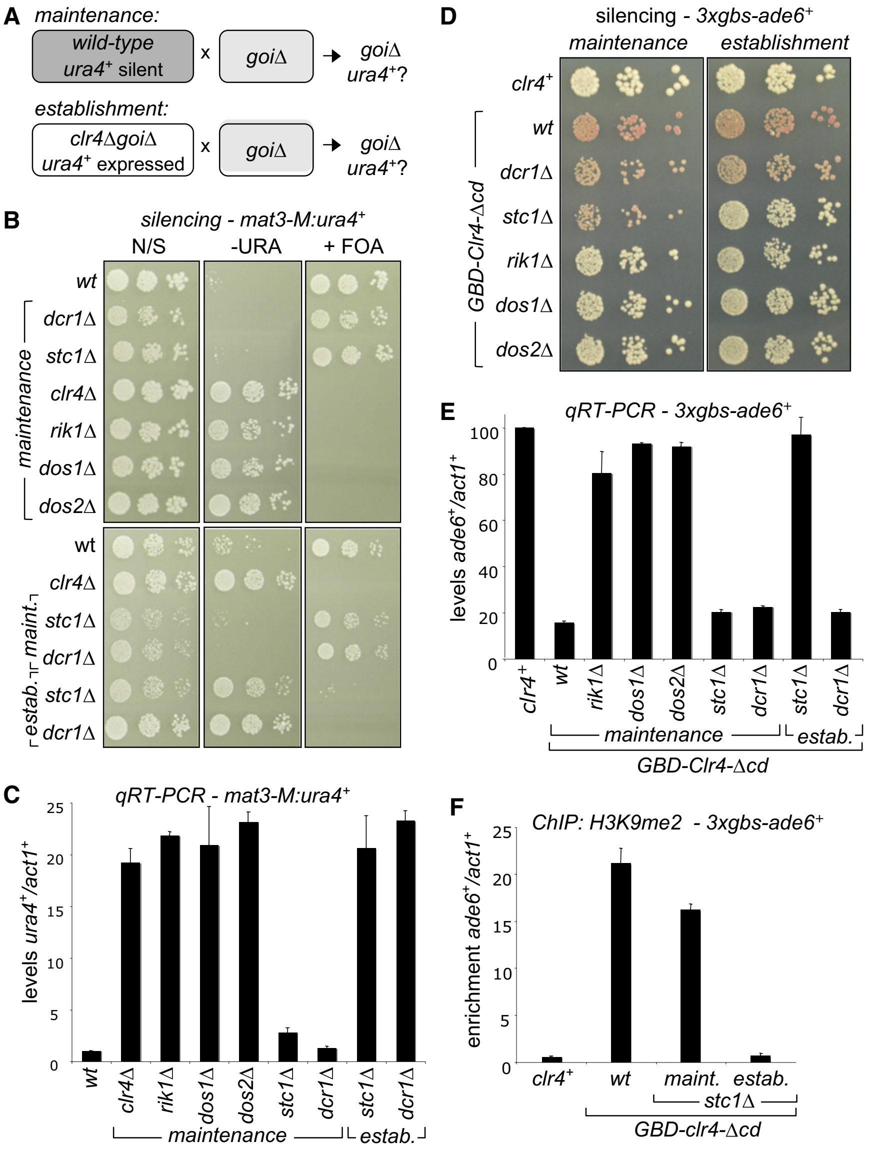

Figure 2. Stc1 Is Required for Establishment but Not Maintenance of Silencing at *mat2-mat3* and a Clr4-Tethered Site

(A) Diagram of crosses performed to assess establishment versus maintenance of silencing of a *ura4*+ marker gene inserted into the mating-type locus (*mat3-M:ura4*+; *goi* is gene of interest).

(B) Assay for silencing at *mat3-M:ura4*+; plates are nonselective (N/S), lacking uracil (−URA) or supplemented with FOA (+FOA). Loss of silencing results in growth on –URA and loss of resistance to FOA.

(C) qRT-PCR analysis of *mat3-M:ura4*+ transcript levels relative to *act1*+, normalized to the wild-type.

(D) Assay for silencing of *3xgbs-ade6*+, mediated by tethered Clr4 (GBD-Clr4-Δcd) (Kagansky et al., 2009). Recruitment of GBD-Clr4 induces silencing of *3xgbs-ade6*+, resulting in red colonies on limiting adenine; loss of silencing leads to white colonies. Maintenance was assessed by crossing mutant cells with cells in which silencing was established; to assess establishment, GBD-Clr4-Δcd and *3xgbs-ade6*+ constructs were combined in mutant backgrounds.

(E) qRT-PCR analysis of *3xgbs-ade6*+ transcript levels relative to *act1*+, normalized to those in cells lacking tethered Clr4 (*clr4*+).

(F) ChIP analysis of H3K9me2 levels on *3xgbs-ade6*+.

See also Figure S2. All error bars indicate SD.

surprisingly, like CLRC components but unlike Dcr1, Stc1 is required for establishment of GBD-Clr4-Δcd-mediated silencing. ChIP analyses confirmed that establishment of H3K9me on the reporter required Stc1, whereas maintenance of H3K9me was largely unaffected in *stc1*Δ cells (Figure 2F). This suggests that, apart from its role in RNAi, Stc1 is additionally required to initiate CLRC activity, even when Clr4 is tethered directly to DNA, although it is not required to sustain this activity once H3K9me is established. This assay reveals a unique role for Stc1 that is distinct from those of other RNAi and CLRC component mutants, suggesting that Stc1 may function at the interface between RNAi and Clr4 methyltransferase activity.

Stc1 Associates with CLRC and RITS Subunits

To gain further insight into the functional relationship between Stc1 and known RNAi and chromatin components, we affinity selected Stc1-FLAG from cell lysates and identified coprecipitating proteins by liquid chromatography-tandem mass spectrometry (LC-MS/MS). Specificity was ensured by subtracting proteins identified in immunoprecipitates (IPs) from untagged control strains (Figure S3A and Tables S3 and S4). Notably, many of the identified peptides corresponded to CLRC components: Cul4, Rik1, Dos1, and Dos2. Nedd8 peptides were also detected, consistent with Cul4 being neddylated (Figure 3A). The association of Stc1-FLAG with Rik1, Dos1, and Dos2 was confirmed by western analysis of IPs to detect Rik1-myc, GFP-Dos1, and Dos2-HA. In addition, although the CLRC component Clr4 was not detected in our LC-MS/MS analysis, association of myc-Clr4 with Stc1-FLAG was detected by IP-western (Figure 3B and Figures S3B and S3C). Interestingly, association of Stc1-FLAG with the RITS component Ago1 (myc-Ago1) was also observed, consistent with the RNAi-like (*dcr1*Δ) phenotype of *stc1*Δ cells (Figure 3C and Figure S3D). Association between Stc1-FLAG and RDRC components was not detected (Figure S3E). The finding that Stc1 associates with both CLRC and Ago1, along with the phenotype of *stc1*Δ cells, indicates that Stc1 may be a key bridging protein that connects the chromatin modification machinery to RNAi.

The association of CLRC subunits Clr4, Rik1, and Dos2 with Stc1-FLAG was next examined in cells lacking RNAi components (*dcr1*Δ or *ago*Δ), Clr4 methyltransferase (*clr4*Δ), or other

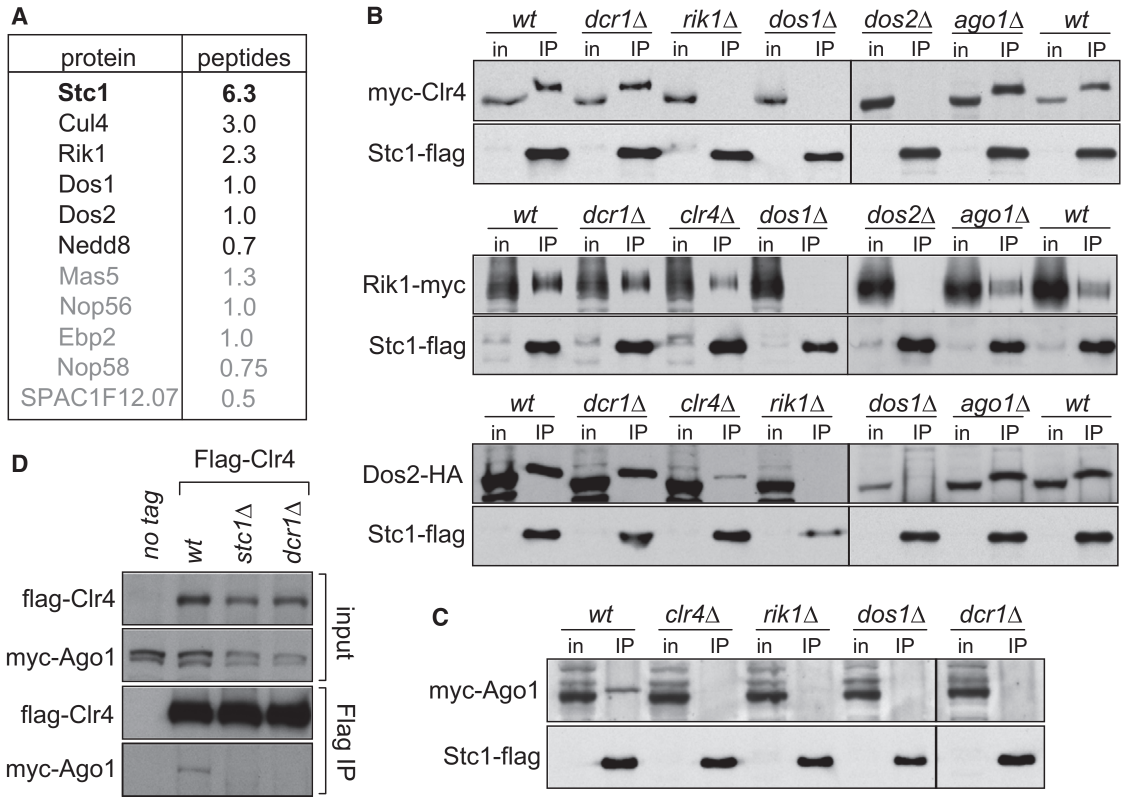

Figure 3. Stc1 Interacts with CLRC and Ago1

(A) List of proteins found specifically and reproducibly associated with Stc1-FLAG by affinity purification and mass spectrometry (LC-MS/MS). Average numbers of peptides identified in each replicate are shown.

(B and C) Stc1-FLAG IP followed by western analysis determining requirements for Stc1 association with Rik1-myc, GFP-Dos1, Dos2-HA, myc-Clr4, and myc-Ago1.

(D) Clr4 association with Ago1 requires Stc1. Flag-Clr4 IP followed by western analysis of myc-Ago1.

See also Figure S3.

CLRC subunits, Rik1 or Dos2 (Figure 3B). IP-western analyses indicated that Clr4, Rik1, and Dos2 associate with Stc1 even in the absence of RNAi (*dcr1*Δ); Stc1 association with CLRC is therefore RNAi independent, and Stc1 may be considered an additional CLRC component. Association of Rik1 and Dos2 with Stc1 is also maintained in *clr4*Δ cells, albeit at reduced levels. However, *dos1*Δ or *dos2*Δ disrupts the association of Clr4 and Rik1 with Stc1, and similarly, *rik1*Δ or *dos1*Δ disrupts association of Dos2 with Stc1. These findings suggest that Rik1, Dos1, and Dos2 are all required for Stc1 association with CLRC; this might be because deletion of any one of these destabilizes the complex. Clr4 is perhaps a more peripheral CLRC component since its absence does not preclude Stc1 association with Rik1 or Dos2. Similarly, deletion of Stc1, which disrupts the association of RITS with RDRC (Figure S3F), does not affect the association of Dos1 with Rik1 and Dos2 in CLRC (Figure S3G). These analyses suggest that Stc1 is not integral to CLRC complex stability, but is an auxiliary component for CLRC.

Similar examination of Stc1 association with Ago1 revealed that this is fully dependent on Clr4, Rik1, and Dos1 (Figure 3C). Strikingly, and in contrast to CLRC, Ago1 association with Stc1-FLAG is also dependent on Dcr1, indicating that Stc1 associates with Ago1 only when RNAi is active. This is consistent with a model in which Stc1 is recruited to sites of active RNAi via Ago1/RITS, bringing with it associated CLRC, thus linking RNAi-based targeting to chromatin modification. In support of this, we detected myc-Ago1 in FLAG-Clr4 immunoprecipitates from wild-type but not *dcr1*Δ or *stc1*Δ cells (Figure 3D). Furthermore, GST-Stc1 and 35S-labeled Ago1 associate with each other, but not with control proteins (^{35S}Cid12 and GST-Scm3), indicating that Stc1 can associate with Ago1 independently of CLRC in vitro (Figure S3H).

RNAi-Dependent Stc1 Recruitment to Outer Repeat Transcripts

We next examined the subcellular localization of GFP-tagged Stc1. In G2 interphase cells, the centromeres cluster, visible as one focus when stained for the centromere-specific H3 variant CENP-A^{Cnp1} (Funabiki et al., 1993; Kniola et al., 2001). Stc1-GFP and CENP-A^{Cnp1} costaining revealed one bright Stc1 focus in close proximity to the CENP-A^{Cnp1} signal, suggesting that Stc1 primarily localizes at centromeres (Figure 4A). Stc1 was lost from centromeres in cells lacking active RNAi (*dcr1*Δ) or

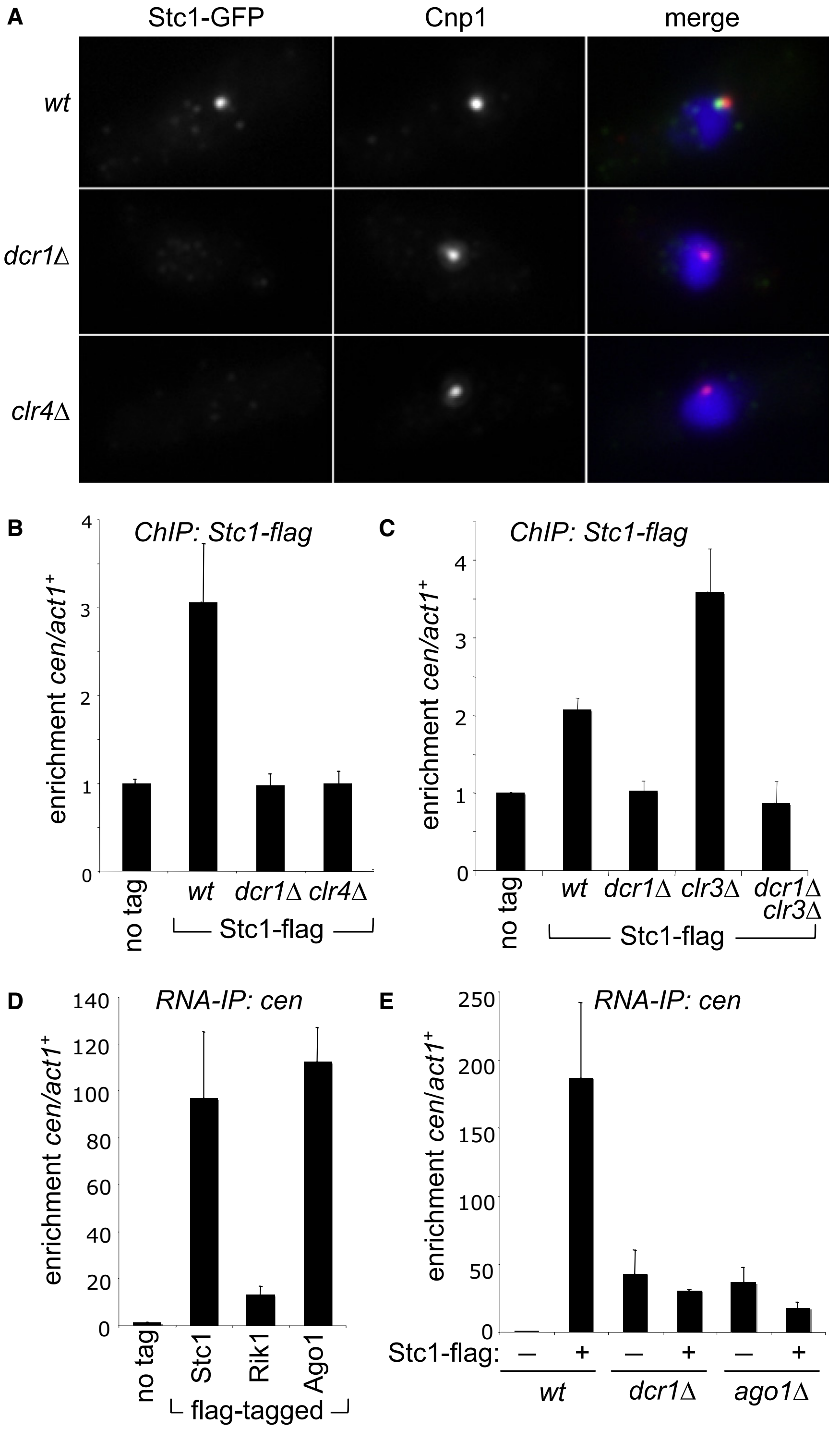

Figure 4. Stc1 Associates with Centromeres and Centromeric Transcripts

(A) Analysis of Stc1-GFP localization in wild-type or mutant cells by immunofluorescence. Representative images show staining of fixed cells for Stc1-GFP (green), Cnp1 (red), and DNA (DAPI—blue).

(B and C) ChIP analysis of Stc1-FLAG association with *cen-dg* relative to $act1^{+}$.

(D and E) RNA immunoprecipitation (RNA-IP) analysis of *cen* transcripts associated with FLAG-tagged Stc1, Rik1, or Ago1 under native conditions. Enrichments shown are normalized to levels of RNA immunoprecipitated from wild-type, untagged control cells; in *dcr1Δ* or *ago1Δ* cells, Stc1-FLAG IPs should be compared to IPs from untagged cells bearing the same mutation since loss of silencing in these mutants causes higher levels of *cen* transcript to accumulate resulting in higher background RNA levels.

See also Figure S4. All error bars indicate SD.

Clr4 methyltransferase (*clr4*Δ). In addition, ChIP analyses confirmed that Stc1-FLAG associates with centromeric outer repeats, dependent on functional Dcr1 and Clr4 (Figure 4B). Thus, like other RNAi-directed chromatin modification components, Stc1 associates with centromeric heterochromatin, and this requires active RNAi and Clr4 methyltransferase. Dcr1- and Clr4-dependent enrichment of Stc1-FLAG at telomeres and *mat2-mat3* was also detected by ChIP (Figure S4A), consistent with localization of other RNAi components at these loci (Cam et al., 2005; Noma et al., 2004).

The histone deacetylase Clr3 suppresses RNAPII transcription in heterochromatin by deacetylating H3K14 (Sugiyama et al., 2007; Wirén et al., 2005; Yamada et al., 2005). Consequently, *clr3*Δ cells exhibit increased levels of heterochromatic transcripts and, because RNAi is not disrupted, greater accumulation of siRNAs. This has been shown to increase recruitment of components engaged in the response to RNAi, such as Rik1 (Zhang et al., 2008). We applied this same test to Stc1, and indeed in *clr3*Δ cells more Stc1-FLAG was detected on centromeric outer repeats, and, as with Rik1, this was dependent on Dcr1 (Figure 4C). Thus, like Rik1, Stc1 association with centromeric heterochromatin is dependent on levels of RNAPII transcripts and siRNAs. This suggests that Stc1 is recruited to centromeres by the action of RNAi on centromeric transcripts.

We next directly tested the association of FLAG-tagged Rik1, Stc1, and Ago1 with centromeric transcripts by IP of RNA from extracts of unfixed cells. Centromeric outer repeat (*dg*) transcripts were enriched ~10-fold relative to $act1^{+}$ gene transcripts in Rik1-FLAG IPs, and ~100-fold in Stc1-FLAG and FLAG-Ago1 IPs (Figure 4D). This may suggest that Stc1 lies in close proximity

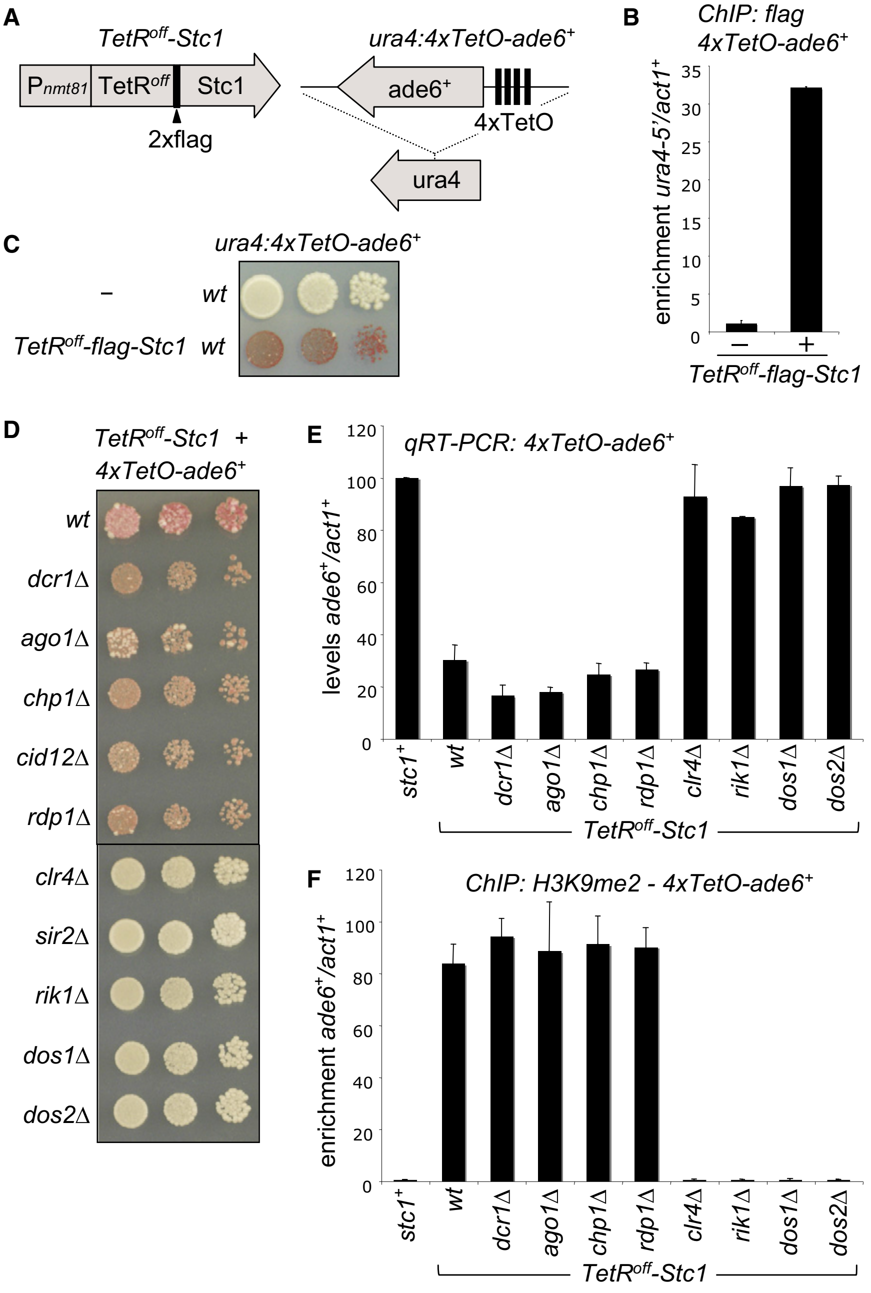

Figure 5. Tethering Stc1 Induces Silencing Independent of RNAi

(A) Diagram of constructs used: *TetR^{off}-Stc1* (integrated at *leu1$^+$*) and the *4xTetO-ade6$^+$* reporter inserted at the *ura4$^+$* locus.

(B) FLAG ChIP analysis of *TetR^{off}-Stc1* fusion protein association with *4xTetO-ade6$^+$* relative to *act1$^+$*.

(C and D) Assay for silencing of *4xTetO-ade6$^+$*. Cells are plated on limiting adenine; red colonies indicate *ade6$^+$* silencing, and white colonies indicate *ade6$^+$* expression.

(E) qRT-PCR analysis of *3xgbs-ade6$^+$* transcript levels relative to *act1$^+$*, normalized to those in cells lacking tethered Stc1 (*stc1$^+$*).

(F) ChIP analysis of H3K9me2 levels on *4xTetO-ade6$^+$*.

All error bars indicate SD.

to noncoding centromeric transcripts, and is consistent with close association of Stc1 with Ago1, which interacts with centromeric transcripts via associated siRNAs. Furthermore, association of Stc1-FLAG with outer repeat transcripts was dependent on Dcr1 and Ago1 (Figure 4E). This confirms that active RNAi and Ago1 are required for Stc1 association with centromeric RNAs and recruitment to cognate chromatin. Stc1 IPs consistently showed greater enrichment for centromeric transcripts than IPs of other CLRC components; this distinction is not due to significant differences in amounts of immunoprecipitated protein (Figures S4B and S4C). Thus, Stc1 and Ago1 are perhaps more closely associated with centromeric transcripts than are Rik1 and other CLRC components. This would be consistent with Stc1 being critical for mediating the interaction of CLRC with transcript-bound RITS, which is supported by the finding that association of Clr4 with Ago1 is dependent on Stc1 (Figure 3D).

Tethered Stc1 Mediates RNAi-Independent Heterochromatin Formation

Stc1 is required to establish RNAi-dependent silencing, and to allow initiation of H3K9 methylation when Clr4 is tethered to DNA, but is not required to maintain H3K9 methylation activity. Thus Stc1 is pivotal for initiating chromatin modification in response to RNAi. If the role of Stc1 is to recruit Clr4/CLRC to chromosomal regions targeted by RNAi, then tethering Stc1 to a euchromatic locus might induce heterochromatin formation independently of RNAi. To test this, Stc1 fused to a TetR^{off} DNA-binding domain plus two FLAG tags (TetR^{off}-Stc1) was expressed in cells with four *tet* operators upstream of an *ade6$^+$* reporter (*4xTetO-ade6$^+$*) (Figure 5A). This resulted in recruitment of TetR^{off}-Stc1 to the reporter and silencing of the *ade6$^+$* gene (Figures 5B and 5C). Notably, maintenance of this TetR^{off}-Stc1-mediated silencing does not require the RNAi components Dcr1, Ago1, Chp1, Cid12, or Rdp1 but is dependent on Clr4, the other CLRC subunits Rik1, Dos1, and Dos2, and the H3K9 deacetylase Sir2 (Figure 5D). qRT-PCR confirmed that TetR^{off}-Stc1-mediated silencing of *4xTetO-ade6$^+$* expression depends on CLRC but not RNAi components (Figure 5E). In addition, ChIP analyses showed that tethered Stc1 induces H3K9 methylation over the reporter that is maintained in RNAi mutants but not in *clr4*Δ, *rik1*Δ, *dos1*Δ, and *dos2*Δ cells (Figure 5F). Normally, Stc1 is

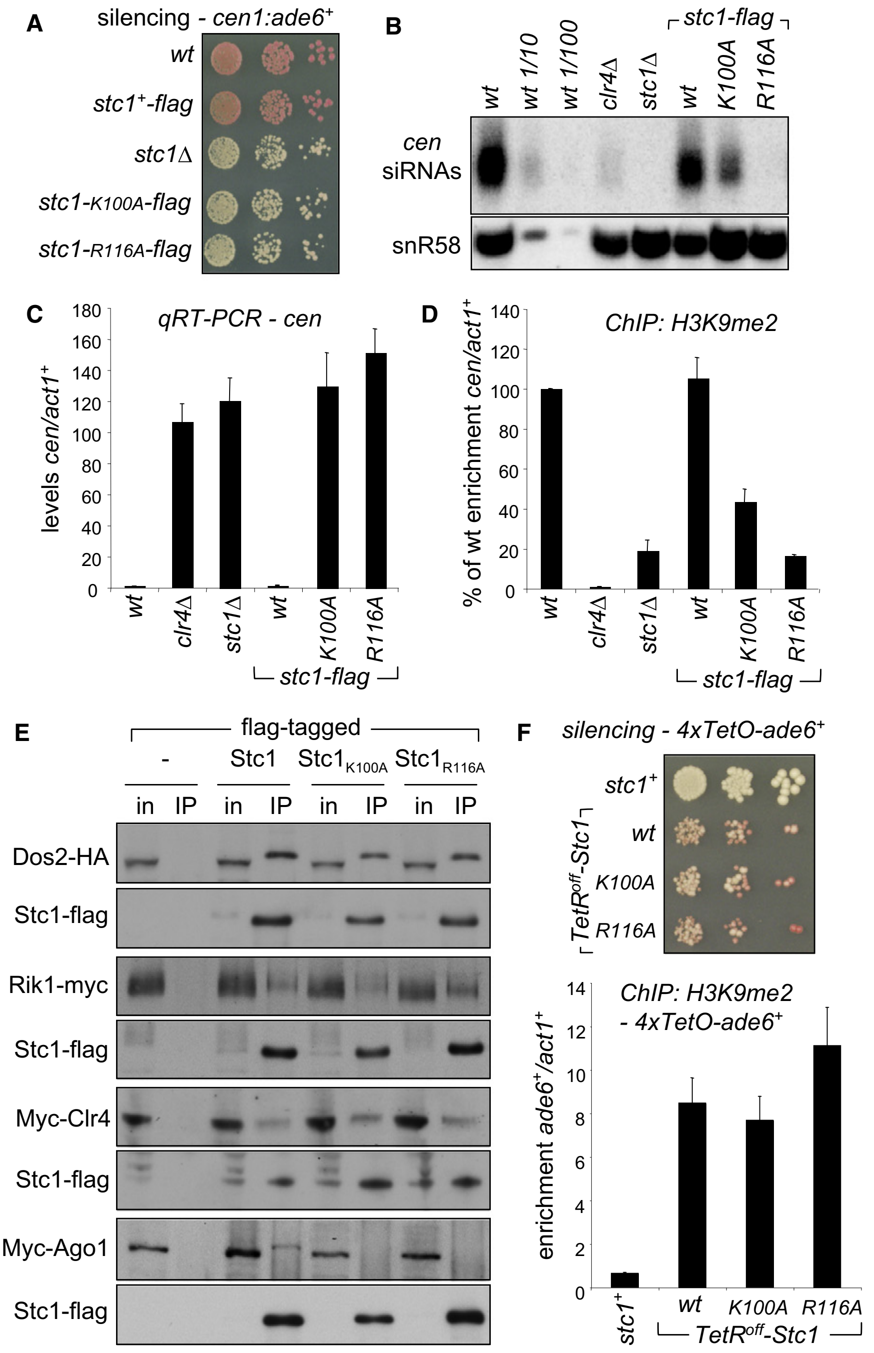

Figure 6. Stc1 Association with Ago1 Is Disrupted by Mutations in the LIM Domain

(A) Assay for silencing of *cen1:ade6*$^+$. Wild-type, silencing cells form red colonies; loss of silencing leads to pink/white colonies.

(B) Northern analysis of centromeric siRNAs, including a dilution series for wild-type. Loading control: snoRNA58 (snR58).

(C) qRT-PCR analysis of *cen(dg)* transcript levels relative to a control transcript *act1*$^+$.

(D) ChIP analysis of H3K9me2 levels associated with *cen(dg)*.

(E) Western analysis of wild-type or mutant Stc1-FLAG IPs to detect Dos2-HA, myc-Clr4, Rik1-myc, or myc-Ago1.

(F) Assay for silencing of *4xTetO-ade6*$^+$ via tethered wild-type or mutant *TetR*^{off}*-Stc1* (red colonies indicate *ade6*$^+$ silencing, white colonies *ade6*$^+$ expression) and ChIP analysis of H3K9me2 levels on *4xTetO-ade6*$^+$.

See also Figure S5. All error bars indicate SD.

recruited to centromeric chromatin in an RNAi-dependent manner, mediated by association with Ago1/RITS. However, this tethering assay indicates that, although required for RNAi, Stc1 can act independently of RNAi to recruit CLRC so as to mediate H3K9 methylation and assemble heterochromatin.

Stc1 Contains a LIM Domain Required for Its Function

stc1$^+$ encodes a 25 kDa protein with no obvious homologs or domains. However, BLAST (Altschul et al., 1997) searches detected proteins homologous to the N-terminal region of Stc1. Iterative similarity searches with this conserved region (Stc1 residues 40–121) using HMMer (Eddy, 1996) identified remote homologs in algae (Figures S5A–S5C). The Stc1 protein family is thus distributed across both fungi and algae but has yet to be found elsewhere. Profile-profile comparisons highlighted a region at the N terminus of Stc1 (residues 40–121) with statistically significant similarity to LIM domains (HHpred probability: 81% and E = 4.2E^{-4} [Söding et al., 2005]). LIM domains possess a tandem zinc-finger (Znf) structure and can mediate protein-protein interactions (Kadrmas and Beckerle, 2004).

To investigate the importance of the LIM domain in Stc1 function, we introduced point mutations into the N-terminal region of FLAG-tagged endogenous *stc1*$^+$. Mutation of core Znf cysteine residues (C83A or C93A) destabilized the Stc1-FLAG protein (data not shown). However, mutation of other conserved LIM domain residues (K100A or R116A) did not affect protein stability but did impair protein function, resulting in defective centromeric silencing similar to that seen in *stc1Δ* cells (Figure 6A and Figures S5D and S5E). Thus, the LIM domain of Stc1 is important for its role in heterochromatin integrity. Further analyses showed that *stc1-K100A* and *stc1-R116A* mutant cells have elevated levels of centromeric transcripts and reduced H3K9 methylation, consistent with defective silencing. Centromeric siRNAs are reduced in *stc1-K100A*, and undetectable in *stc1-R116A*, cells (Figures 6B–6D). However, IP-western analyses indicated that both Stc1^{K100A} and Stc1^{R116A} proteins still associate with Clr4, Rik1 and Dos2,

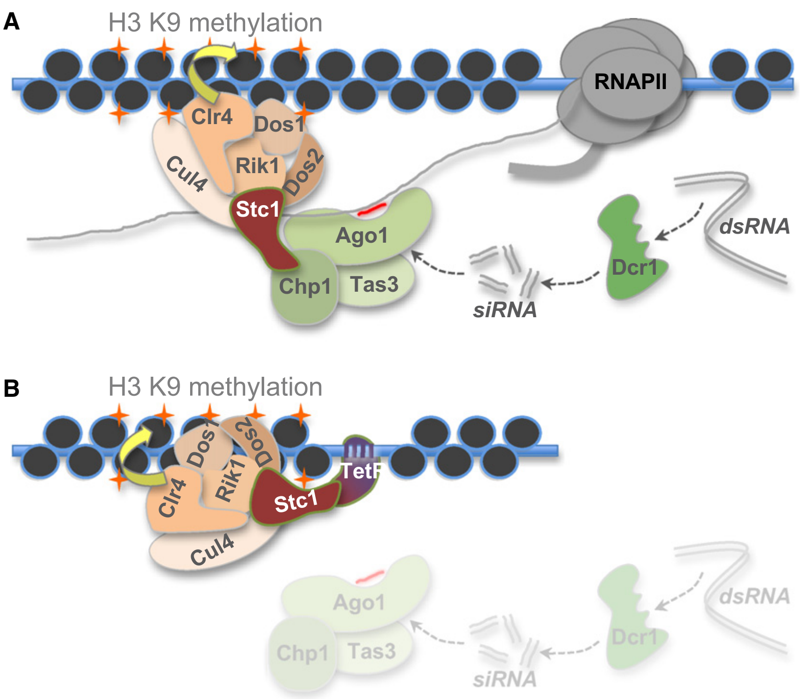

Figure 7. Model for the Function of Stc1

(A) Dcr1-generated siRNAs associate with Ago1 and guide RITS to homologous nascent transcripts. Once RITS has engaged these transcripts it recruits Stc1 via the LIM domain, which mediates the association of Clr4 methyltransferase activity/CLRC. In this way, Stc1 connects the chromatin modification machinery with the siRNA targeting signal, facilitating RNAi-directed H3K9 methylation and heterochromatin formation.

(B) Tethering Stc1 to DNA via a DNA-binding domain fusion bypasses the requirement for the siRNA-dependent targeting of RITS to nascent transcripts that normally mediates localization of Stc1 to chromatin. Thus, in this system Stc1 can recruit Clr4 methyltransferase activity to target chromatin independent of active RNAi and of its association with Ago1/RITS.

indicating that LIM domain mutations do not affect Stc1-CLRC interactions (Figure 6E). In contrast, we could not detect association of Stc1^{K100A} or Stc1^{R116A} proteins with Ago1, suggesting that it is the ability of Stc1 to couple CLRC to Ago1/RNAi that is specifically impaired in these mutants. To test this, we assessed the ability of Stc1^{K100A} and Stc1^{R116A} to promote CLRC activity and establish silencing independently of RNAi, at a tethered locus. As with wild-type Stc1, recruitment of Stc1^{K100A} or Stc1^{R116A} to *ade6*$^{+}$ by fusion to TetR^{off} resulted in H3K9 methylation and silencing (Figure 6F). Consistent with this, establishment of silencing by tethered Clr4, which requires Stc1 (Figure 2), was unaffected by these Stc1 LIM domain mutations (Figures S5F and S5G). Together, these assays reveal that mutation of the LIM domain does not affect the ability of Stc1 to recruit and promote CLRC activity, but does affect the interaction of Stc1 with Ago1/RITS. Thus, by uncoupling the functions of Stc1 in associating with Ago1/RITS, and recruiting CLRC, these analyses support a pivotal role for Stc1 in connecting these two complexes to facilitate heterochromatin formation.

DISCUSSION

There is currently a significant gap in our understanding of how RNAi directs chromatin modifications that result in heterochromatin formation and transcriptional silencing. The consensus view of RNAi-mediated chromatin modification in fission yeast is that siRNAs guide the RITS complex to homologous nascent transcripts, and this triggers events that lead to chromatin modification (Bühler and Moazed, 2007; Grewal and Jia, 2007). However, it is not known how transcript-associated RITS recruits the key chromatin modifier Clr4 to induce the formation of heterochromatin. Here, we have identified a bridging protein, Stc1, with a pivotal role in integrating cotranscriptional RNAi with chromatin modification.

The conclusion that Stc1 provides a critical link between RNAi and the chromatin-modifying CLRC complex is supported by several lines of evidence. First, the limited effects of the *stc1Δ* mutant on maintenance of H3K9 methylation at centromeres and *mat2-mat3* resemble RNAi mutants and indicate that Stc1 is not required for all H3K9 methylation activity, but is specifically required for RNAi-directed heterochromatin formation. Second, Stc1 associates with Ago1 in vivo and in vitro, and, like Ago1, it associates with outer repeat transcripts, dependent on active RNAi, and is required for siRNA accumulation. Third, Stc1 also associates with components of the CLRC complex, including Clr4, and this occurs independently of RNAi. Fourth, association of Ago1 with Clr4 is dependent on Stc1. Fifth, when tethered to a euchromatic locus, Stc1 can induce heterochromatin formation independently of Ago1 and RNAi. Thus, Stc1 is sufficient to recruit Clr4/CLRC activities, allowing them to modify local chromatin to effect heterochromatin formation. Finally, a mutation in the LIM domain of Stc1 does not affect its ability to recruit CLRC and initiate heterochromatin when tethered to DNA, but does disrupt its association with Ago1 and the integrity of centromeric heterochromatin. This indicates that association with Ago1 and recruitment/activation of CLRC are distinct activities of Stc1, both of which are essential for RNAi-directed heterochromatin formation. Together, these analyses suggest that normally, when Ago1 is guided by an siRNA to a target nascent transcript, it is the recruitment of Stc1 that mediates the association of CLRC, allowing Clr4 to methylate H3K9 in nearby cognate chromatin (Figure 7).

It has previously been shown that CLRC components Rik1 and Clr4 associate with Chp1, a component of RITS (Zhang et al., 2008). Both Stc1 and Rik1 are recruited to centromeres in response to outer repeat transcription and siRNA production, and both are associated with noncoding centromeric transcripts. However, 10-fold more transcript associates with Stc1 than with Rik1, suggesting that Stc1 might be more closely associated with centromeric outer repeat RNA. This is consistent with the observed association of Stc1 with Ago1, which interacts with

centromeric transcripts via associated siRNAs. In addition, tethering experiments demonstrate that Rik1 and Clr4 are required for Stc1-mediated silencing, whereas Stc1 (but not Rik1) is dispensable for maintenance of Clr4 mediated silencing. These findings point to an order of events in which Stc1 functions upstream of Rik1 and the other CLRC components, mediating their delivery to sites of active RNAi, but being dispensable for ongoing chromatin modification.

Stc1 associates with members of the CLRC complex independently of active RNAi. However, Stc1 differs from other CLRC components in that it is not required to maintain all CLRC activity: maintenance of H3K9 methylation at *mat2-mat3*, and at a site silenced by tethered Clr4, is independent of RNAi and is unaffected by Stc1 deletion. The finding that Rik1, Dos1, and Dos2 remain associated in the absence of Stc1 is consistent with Stc1 being dispensable for ongoing CLRC activity at some loci and suggests that Stc1 may not be a core CLRC component. Rather, the role of Stc1 appears to be that of an adaptor protein for the CLRC complex that mediates its interaction with the RNAi machinery (Figure 7). This is supported by our finding that, unlike other CLRC components, deletion of Stc1 abolishes detectable siRNA accumulation. This indicates that Stc1 is more than an auxiliary component for CLRC: it is also intimately associated with, and required for, RNAi. It may be that, in addition to facilitating CLRC recruitment, association of Stc1 with Ago1 is important for Ago1 function. It is also possible that Stc1 contributes to a positive feedback mechanism mediating recruitment of RNAi to sites of H3K9me, thus propagating H3K9me to form robust heterochromatin.

The finding that Stc1 is required for establishment of silencing by tethered Clr4, which is RNAi independent, suggests that Stc1 has an additional role in initiating CLRC activity after Clr4 recruitment. Stc1 may promote the association of other CLRC components with Clr4 or facilitate a CLRC/Clr4 activation step. Apart from the methyltransferase Clr4, the CLRC complex contains Rik1, Dos1, Dos2, and Cul4, which, together with associated Rbx1 and Nedd8, comprise an active Cullin-dependent E3 ubiquitin ligase (Hong et al., 2005; Horn et al., 2005; Jia et al., 2005; Thon et al., 2005; Zhang et al., 2008). The key substrates of this E3 ligase remain unclear; however, one possibility is that ubiquitination of target site chromatin renders histone H3 accessible for H3K9 methylation, analogous to the promotion of H3K79 methylation by H2BK123 monoubiquitination in *S. cerevisiae* (Briggs et al., 2002; Ng et al., 2002). Consistent with this possibility, CLRC has been shown to polyubiquitinate histone H2B in vitro, and ubiquitinated proteins similar in size to histones were detected in CLRC purifications (Hong et al., 2005; Horn et al., 2005). Such ubiquitination could be a key event allowing the formation of heterochromatin, potentially requiring Stc1. Alternatively, Stc1 might promote the histone deacetylation that is required to permit H3K9 methylation (Nakayama et al., 2001; Shankaranarayana et al., 2003).

Despite the discovery of many of factors contributing to RNAi-directed heterochromatin formation in fission yeast, the mechanism by which active RNAi recruits chromatin modifiers has remained enigmatic (reviewed in Bühler and Moazed, 2007; Grewal and Jia, 2007). One reason for this is the inherent codependency among components of the system, which makes it difficult to determine the order in which they operate. A solution to this problem is to reconstitute heterochromatin at euchromatic loci. This can be achieved by introducing an artificial source of siRNAs that target RNAi to a euchromatic gene (Iida et al., 2008) or by directly tethering RNAi factors or chromatin modifiers to a target locus (Bühler et al., 2006). We have previously shown that tethering Clr4 to a reporter gene by means of a DNA-binding domain bypasses the requirement for RNAi in heterochromatin formation (Kagansky et al., 2009). Here, we find that in *stc1Δ* cells, silencing mediated by tethered Clr4 can be maintained, but cannot be established. This unique phenotype reveals that Stc1 is required specifically to initiate Clr4 activity at the target locus. Similar tethering experiments with Stc1 allowed us to demonstrate that it can also act independently of RNAi to recruit Clr4. These observations are consistent with the findings that RNAi (Hall et al., 2002), and now Stc1, are critical for establishment, but play only a minor role in maintenance, of heterochromatin at the silent mating-type locus. This is because DNA-binding proteins act at this locus to maintain heterochromatin after its establishment (Jia et al., 2004; Kim et al., 2004). Recruitment of Clr4 methyltransferase activity by tethering it either directly or via Stc1 essentially recapitulates this form of RNAi-independent silencing in a way that is more easily dissected.

Stc1 contains a LIM domain that is required for its association with Ago1, and hence its role in heterochromatin formation. LIM domain proteins are found in a wide range of eukaryotes from yeast to humans and are generally involved in mediating protein-protein interactions that can have key roles in integrating biological circuits (reviewed in Kadrmas and Beckerle, 2004). Consistent with this, we propose that the role of Stc1 is to integrate the RNAi and chromatin modification pathways. RNAi-directed chromatin modification is widespread in plants (Matzke et al., 2009), and recent evidence suggests that germline-associated small RNAs called Piwi-interacting RNAs (piRNAs) may mediate silencing via chromatin modification in animals (Klenov et al., 2007). We have shown that Stc1 is critical for recruitment of the Clr4 H3K9 methyltransferase to sites of active RNAi in fission yeast; LIM domain proteins distantly related to Stc1 could have conserved roles in coupling noncoding RNAs to chromatin modification in other systems.

EXPERIMENTAL PROCEDURES

Yeast Strains and Plasmids

For *S. pombe* strains, see Table S1. Deletion and epitope tagging (3xFLAG or GFP) of Stc1 were achieved by homologous recombination with PCR fragments comprising resistance cassettes flanked by sequence homologous to insertion sites (Bähler et al., 1998). LIM domain mutations were generated with Quickchange II XL Kit (Stratagene) and integrated by replacement of *stc1:ura4*$^{+}$. The Clr4-GBD tethering system was described previously (Kagansky et al., 2009). For pDUAL-TetR-2xFLAG-Stc1, *stc1*$^{+}$ was cloned into a modified pDUAL-HFF81c vector (Matsuyama et al., 2004), made by swapping His to TetR^{off} (Promega), so that Stc1 was N-terminally tagged with TetR^{off} followed by 2xFLAG, under the *nmt81* promoter. Not1-digested plasmid was integrated at *leu1*$^{+}$. For the *4xTetO-ade6*$^{+}$ reporter, 4xTetO repeats (TCCCTATCAGTGATAGAGA) were introduced upstream of *ade6*$^{+}$ inserted at the *ura4*$^{+}$ locus via the strategy described previously for *3xgbs-ade6*$^{+}$ (Kagansky et al., 2009).

ChIP

ChIP was performed as described (Pidoux et al., 2004) with the following modifications: Cells were fixed in 1% PFA/15 min for H3K9me2 or Stc1-FLAG ChIP, or 3% PFA/30 min for Swi6 ChIP. For Swi6 and Stc1-FLAG ChIPs, cells were incubated for 2 hr at 18°C prior to fixing. One microliter monoclonal H3K9me2 antibody (m5.1.1) (Nakagawachi et al., 2003), 10 μl polyclonal Swi6 antibody (Ekwall et al., 1995) or 1 μl anti-Flag M2 antibody (Sigma, F1804) were used per ChIP. qPCR analysis primers are in Table S2. Relative enrichments were calculated as the ratio of product of interest to control product (*act1*$^{+}$) in IP over input. Histograms represent data from three biological replicates analyzed in parallel.

Immunoaffinity Purification

Immunoaffinity purifications for LC-MS/MS analysis were performed as described (Oeffinger et al., 2007), with the following modifications: IPs were performed on 5 g of cells using Dynabeads coupled to anti-Flag M2 antibody (Sigma, F1804) for 15 min. The IP'd material was treated with 500 U Benzonase, washed, subjected to on-bead Tryptic digestion, and prepared for LC-MS/MS analysis as described previously (Bayne et al., 2008). Figure 3A lists proteins identified in at least two out of four independent purifications of Stc1-flag and absent from control purifications from untagged cells. Ribosomal proteins (common contaminants) were also excluded.

RNA-IPs were performed as above but with the addition of superRNAsin (Ambion) to the lysis buffer and excluding Benzonase. After washes, immunoprecipitated material was resuspended in RNA extraction buffer (25 mM Tris-HCl [pH 7.5], 5 mM EDTA [pH 8], 50 mM NaCl, 0.5% SDS) with 200 ng/ml proteinase K and incubated at 37°C for 2 hr. RNA was extracted with phenol: chloroform, precipitated with ethanol and 1 μl glycogen, and analyzed by qRT-PCR.

Co-IPs for western analysis were performed on 2 g of cells as above but for 1 hr. IPed material was washed four times with lysis buffer, resuspended in SDS sample buffer, and analyzed by SDS-PAGE. Antibodies used were anti-flag M2 (Sigma), anti-HA 12CA5 (K. Samejima), anti-GFP (K. Hardwick), and anti-myc 9E10 (Covance), all at 1:1000.

In Vitro Binding Assays

Recombinant protein was expressed from pDEST15 and purified. 35S-labeled protein was produced with a TNT T7 kit (Promega). For in vitro binding, 4 μg GST fusion protein was added to glutathione-agarose on ice for 30 min in binding buffer (50 mM HEPES [pH 7.6], 75 mM KCl, 1 mM MgCl2, 0.5 M NaCl, 0.5 mM DTT, 5 mg/ml BSA, 0.5% NP-40, 1 mM PMSF, Protease inhibitors), then incubated with 10 μl 35S-labeled protein for 1 hr followed by four washes in binding buffer. Samples were analyzed by SDS-PAGE and fluorography.

RNA Analysis

Northern analysis of centromeric siRNAs and qRT-PCR analysis of transcripts were performed as described previously (Bayne et al., 2008). siRNA probes and primers for qRT-PCR are in Table S2.

qPCR

qPCR was performed with SYBR Green on a Bio-Rad iCycler and analyzed with iCycler iQ Optical System Software. Histograms represent three biological replicates; error bars represent one standard deviation.

Cytology

Immunolocalization was performed as described previously (Pidoux et al., 2009). Cells were fixed with 3.7% PFA/10 min, plus 0.05% glutaraldehyde for tubulin staining. Antibodies used were TAT1 anti-tubulin 1:15 (K. Gull), anti-Cnp1 1:1000, and anti-GFP 1:200 (Molecular Probes). Alexa Fluor 594- and 488-coupled secondary antibodies were used at 1:1000 (Invitrogen).

SUPPLEMENTAL INFORMATION

Supplemental Information includes five figures and four tables and can be found with this article online at doi:10.1016/j.cell.2010.01.038.

ACKNOWLEDGMENTS

We thank Allshire Lab members for advice, F. Alves for mass spectrometry support, and J. Hayles for deriving the haploid deletion collection. We are grateful to T. Urano for provision of anti-H3K9me2 and Z. Cande, D. Moazed, and J. Partridge for strains. This research was supported by the Epigenome Network of Excellence (EC-FP6/Contract/LSHG-CT-2004-503433 to R.C.A.), an EC Marie-Curie Excellence grant (MEXT-CT-014171 to J.R.); EMBO-LTF (ALT325-2008 to L.S.P.), an MRC Strategic grant (G0301153 to R.C.A.), MRC-FGU Core Support (to C.P.P.), Wellcome Trust Senior Research fellowship (084229/Z/07/Z to J.R.); Wellcome Trust Programme grant (065061/Z to R.C.A.). R.C.A. is a Wellcome Trust Principal Research Fellow.

Received: July 28, 2009
Revised: November 23, 2009
Accepted: January 21, 2010
Published: March 4, 2010

REFERENCES

Allshire, R.C., Nimmo, E.R., Ekwall, K., Javerzat, J.P., and Cranston, G. (1995). Mutations derepressing silent centromeric domains in fission yeast disrupt chromosome segregation. Genes Dev. *9*, 218–233.

Altschul, S.F., Madden, T.L., Schäffer, A.A., Zhang, J., Zhang, Z., Miller, W., and Lipman, D.J. (1997). Gapped BLAST and PSI-BLAST: a new generation of protein database search programs. Nucleic Acids Res. *25*, 3389–3402.

Bähler, J., Wu, J.Q., Longtine, M.S., Shah, N.G., McKenzie, A., 3rd, Steever, A.B., Wach, A., Philippsen, P., and Pringle, J.R. (1998). Heterologous modules for efficient and versatile PCR-based gene targeting in Schizosaccharomyces pombe. Yeast *14*, 943–951.

Bannister, A.J., Zegerman, P., Partridge, J.F., Miska, E.A., Thomas, J.O., Allshire, R.C., and Kouzarides, T. (2001). Selective recognition of methylated lysine 9 on histone H3 by the HP1 chromo domain. Nature *410*, 120–124.

Bayne, E.H., Portoso, M., Kagansky, A., Kos-Braun, I.C., Urano, T., Ekwall, K., Alves, F., Rappsilber, J., and Allshire, R.C. (2008). Splicing factors facilitate RNAi-directed silencing in fission yeast. Science *322*, 602–606.

Bernstein, E., and Allis, C.D. (2005). RNA meets chromatin. Genes Dev. *19*, 1635–1655.

Briggs, S.D., Xiao, T.J., Sun, Z.W., Caldwell, J.A., Shabanowitz, J., Hunt, D.F., Allis, C.D., and Strahl, B.D. (2002). Gene silencing: trans-histone regulatory pathway in chromatin. Nature *418*, 498.

Bühler, M., and Moazed, D. (2007). Transcription and RNAi in heterochromatic gene silencing. Nat. Struct. Mol. Biol. *14*, 1041–1048.

Bühler, M., Verdel, A., and Moazed, D. (2006). Tethering RITS to a nascent transcript initiates RNAi- and heterochromatin-dependent gene silencing. Cell *125*, 873–886.

Cam, H.P., Sugiyama, T., Chen, E.S., Chen, X., FitzGerald, P.C., and Grewal, S.I.S. (2005). Comprehensive analysis of heterochromatin- and RNAi-mediated epigenetic control of the fission yeast genome. Nat. Genet. *37*, 809–819.

Eddy, S.R. (1996). Hidden Markov models. Curr. Opin. Struct. Biol. *6*, 361–365.

Ekwall, K., Javerzat, J.P., Lorentz, A., Schmidt, H., Cranston, G., and Allshire, R. (1995). The chromodomain protein Swi6: a key component at fission yeast centromeres. Science *269*, 1429–1431.

Ekwall, K., Nimmo, E.R., Javerzat, J.P., Borgstrøm, B., Egel, R., Cranston, G., and Allshire, R. (1996). Mutations in the fission yeast silencing factors clr4+ and rik1+ disrupt the localisation of the chromo domain protein Swi6p and impair centromere function. J. Cell Sci. *109*, 2637–2648.

Funabiki, H., Hagan, I., Uzawa, S., and Yanagida, M. (1993). Cell cycle-dependent specific positioning and clustering of centromeres and telomeres in fission yeast. J. Cell Biol. *121*, 961–976.

Ghildiyal, M., and Zamore, P.D. (2009). Small silencing RNAs: an expanding universe. Nat. Rev. Genet. *10*, 94–108.

Grewal, S.I., and Jia, S. (2007). Heterochromatin revisited. Nat. Rev. Genet. *8*, 35–46.

Grewal, S.I., Bonaduce, M.J., and Klar, A.J. (1998). Histone deacetylase homologs regulate epigenetic inheritance of transcriptional silencing and chromosome segregation in fission yeast. Genetics *150*, 563–576.

Hall, I.M., Shankaranarayana, G.D., Noma, K., Ayoub, N., Cohen, A., and Grewal, S.I. (2002). Establishment and maintenance of a heterochromatin domain. Science *297*, 2232–2237.

Hall, I.M., Noma, K., and Grewal, S.I.S. (2003). RNA interference machinery regulates chromosome dynamics during mitosis and meiosis in fission yeast. Proc. Natl. Acad. Sci. USA *100*, 193–198.

Hansen, K.R., Ibarra, P.T., and Thon, G. (2006). Evolutionary-conserved telomere-linked helicase genes of fission yeast are repressed by silencing factors, RNAi components and the telomere-binding protein Taz1. Nucleic Acids Res. *34*, 78–88.

Hong, E.J., Villén, J., Gerace, E.L., Gygi, S.P., and Moazed, D. (2005). A cullin E3 ubiquitin ligase complex associates with Rik1 and the Clr4 histone H3-K9 methyltransferase and is required for RNAi-mediated heterochromatin formation. RNA Biol. *2*, 106–111.

Horn, P.J., Bastie, J.N., and Peterson, C.L. (2005). A Rik1-associated, cullin-dependent E3 ubiquitin ligase is essential for heterochromatin formation. Genes Dev. *19*, 1705–1714.

Iida, T., Nakayama, J., and Moazed, D. (2008). siRNA-mediated heterochromatin establishment requires HP1 and is associated with antisense transcription. Mol. Cell *31*, 178–189.

Jia, S., Noma, K., and Grewal, S.I. (2004). RNAi-independent heterochromatin nucleation by the stress-activated ATF/CREB family proteins. Science *304*, 1971–1976.

Jia, S., Kobayashi, R., and Grewal, S.I. (2005). Ubiquitin ligase component Cul4 associates with Clr4 histone methyltransferase to assemble heterochromatin. Nat. Cell Biol. *7*, 1007–1013.

Kadrmas, J.L., and Beckerle, M.C. (2004). The LIM domain: from the cytoskeleton to the nucleus. Nat. Rev. Mol. Cell Biol. *5*, 920–931.

Kagansky, A., Folco, H.D., Almeida, R., Pidoux, A.L., Boukaba, A., Simmer, F., Urano, T., Hamilton, G.L., and Allshire, R.C. (2009). Synthetic heterochromatin bypasses RNAi and centromeric repeats to establish functional centromeres. Science *324*, 1716–1719.

Kanoh, J., Sadaie, M., Urano, T., and Ishikawa, F. (2005). Telomere binding protein Taz1 establishes Swi6 heterochromatin independently of RNAi at telomeres. Curr. Biol. *15*, 1808–1819.

Kim, H.S., Choi, E.S., Shin, J.A., Jang, Y.K., and Park, S.D. (2004). Regulation of Swi6/HP1-dependent heterochromatin assembly by cooperation of components of the mitogen-activated protein kinase pathway and a histone deacetylase Clr6. J. Biol. Chem. *279*, 42850–42859.

Klenov, M.S., Lavrov, S.A., Stolyarenko, A.D., Ryazansky, S.S., Aravin, A.A., Tuschl, T., and Gvozdev, V.A. (2007). Repeat-associated siRNAs cause chromatin silencing of retrotransposons in the Drosophila melanogaster germline. Nucleic Acids Res. *35*, 5430–5438.

Kloc, A., and Martienssen, R. (2008). RNAi, heterochromatin and the cell cycle. Trends Genet. *24*, 511–517.

Kniola, B., O'Toole, E., McIntosh, J.R., Mellone, B., Allshire, R., Mengarelli, S., Hultenby, K., and Ekwall, K. (2001). The domain structure of centromeres is conserved from fission yeast to humans. Mol. Biol. Cell *12*, 2767–2775.

Li, F., Goto, D.B., Zaratiegui, M., Tang, X., Martienssen, R., and Cande, W.Z. (2005). Two novel proteins, dos1 and dos2, interact with rik1 to regulate heterochromatic RNA interference and histone modification. Curr. Biol. *15*, 1448–1457.

Matsuyama, A., Shirai, A., Yashiroda, Y., Kamata, A., Horinouchi, S., and Yoshida, M. (2004). pDUAL, a multipurpose, multicopy vector capable of chromosomal integration in fission yeast. Yeast *21*, 1289–1305.

Matzke, M., Kanno, T., Daxinger, L., Huettel, B., and Matzke, A.J. (2009). RNA-mediated chromatin-based silencing in plants. Curr. Opin. Cell Biol. *21*, 367–376.

Motamedi, M.R., Verdel, A., Colmenares, S.U., Gerber, S.A., Gygi, S.P., and Moazed, D. (2004). Two RNAi complexes, RITS and RDRC, physically interact and localize to noncoding centromeric RNAs. Cell *119*, 789–802.

Nakagawachi, T., Soejima, H., Urano, T., Zhao, W., Higashimoto, K., Satoh, Y., Matsukura, S., Kudo, S., Kitajima, Y., Harada, H., et al. (2003). Silencing effect of CpG island hypermethylation and histone modifications on O6-methylguanine-DNA methyltransferase (MGMT) gene expression in human cancer. Oncogene *22*, 8835–8844.

Nakayama, J., Rice, J.C., Strahl, B.D., Allis, C.D., and Grewal, S.I. (2001). Role of histone H3 lysine 9 methylation in epigenetic control of heterochromatin assembly. Science *292*, 110–113.

Neuwald, A.F., and Poleksic, A. (2000). PSI-BLAST searches using hidden markov models of structural repeats: prediction of an unusual sliding DNA clamp and of beta-propellers in UV-damaged DNA-binding protein. Nucleic Acids Res. *28*, 3570–3580.

Ng, H.H., Xu, R.M., Zhang, Y., and Struhl, K. (2002). Ubiquitination of histone H2B by Rad6 is required for efficient Dot1-mediated methylation of histone H3 lysine 79. J. Biol. Chem. *277*, 34655–34657.

Noma, K., Sugiyama, T., Cam, H., Verdel, A., Zofall, M., Jia, S.T., Moazed, D., and Grewal, S.I.S. (2004). RITS acts in cis to promote RNA interference-mediated transcriptional and post-transcriptional silencing. Nat. Genet. *36*, 1174–1180.

Oeffinger, M., Wei, K.E., Rogers, R., DeGrasse, J.A., Chait, B.T., Aitchison, J.D., and Rout, M.P. (2007). Comprehensive analysis of diverse ribonucleoprotein complexes. Nat. Methods *4*, 951–956.

Pidoux, A., Mellone, B., and Allshire, R. (2004). Analysis of chromatin in fission yeast. Methods *33*, 252–259.

Pidoux, A.L., Choi, E.S., Abbott, J.K.R., Liu, X.K., Kagansky, A., Castillo, A.G., Hamilton, G.L., Richardson, W., Rappsilber, J., He, X., and Allshire, R.C. (2009). Fission yeast Scm3: A CENP-A receptor required for integrity of subkinetochore chromatin. Mol. Cell *33*, 299–311.

Sadaie, M., Iida, T., Urano, T., and Nakayama, J. (2004). A chromodomain protein, Chp1, is required for the establishment of heterochromatin in fission yeast. EMBO J. *23*, 3825–3835.

Shankaranarayana, G.D., Motamedi, M.R., Moazed, D., and Grewal, S.I. (2003). Sir2 regulates histone H3 lysine 9 methylation and heterochromatin assembly in fission yeast. Curr. Biol. *13*, 1240–1246.

Söding, J., Biegert, A., and Lupas, A.N. (2005). The HHpred interactive server for protein homology detection and structure prediction. Nucleic Acids Res. *33* (*Web Server issue*), W244–W248.

Sugiyama, T., Cam, H.P., Sugiyama, R., Noma, K., Zofall, M., Kobayashi, R., and Grewal, S.I.S. (2007). SHREC, an effector complex for heterochromatic transcriptional silencing. Cell *128*, 491–504.

Thon, G., Hansen, K.R., Altes, S.P., Sidhu, D., Singh, G., Verhein-Hansen, J., Bonaduce, M.J., and Klar, A.J. (2005). The Clr7 and Clr8 directionality factors and the Pcu4 cullin mediate heterochromatin formation in the fission yeast Schizosaccharomyces pombe. Genetics *171*, 1583–1595.

Verdel, A., Jia, S., Gerber, S., Sugiyama, T., Gygi, S., Grewal, S.I., and Moazed, D. (2004). RNAi-mediated targeting of heterochromatin by the RITS complex. Science *303*, 672–676.

Volpe, T.A., Kidner, C., Hall, I.M., Teng, G., Grewal, S.I., and Martienssen, R.A. (2002). Regulation of heterochromatic silencing and histone H3 lysine-9 methylation by RNAi. Science *297*, 1833–1837.

Volpe, T., Schramke, V., Hamilton, G.L., White, S.A., Teng, G., Martienssen, R.A., and Allshire, R.C. (2003). RNA interference is required for normal centromere function in fission yeast. Chromosome Res. *11*, 137–146.

Wirén, M., Silverstein, R.A., Sinha, I., Walfridsson, J., Lee, H.M., Laurenson, P., Pillus, L., Robyr, D., Grunstein, M., and Ekwall, K. (2005). Genomewide analysis of nucleosome density histone acetylation and HDAC function in fission yeast. EMBO J. *24*, 2906–2918.

Yamada, T., Fischle, W., Sugiyama, T., Allis, C.D., and Grewal, S.I. (2005). The nucleation and maintenance of heterochromatin by a histone deacetylase in fission yeast. Mol. Cell *20*, 173–185.

Zhang, K., Mosch, K., Fischle, W., and Grewal, S.I. (2008). Roles of the Clr4 methyltransferase complex in nucleation, spreading and maintenance of heterochromatin. Nat. Struct. Mol. Biol. *15*, 381–388.

Cell

A mini-chromosome (*Ch16*) loss rate

genotype	total colonies	half-sectored colonies	% loss
wt	1360	1	**0.07**
clr4Δ	1279	36	**2.81**
stc1Δ	1183	32	**2.70**

B

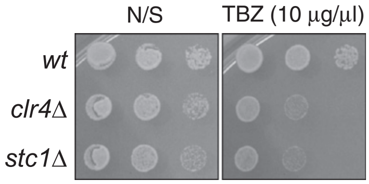

Figure S1. Deletion of Stc1 Results in High Rates of Minichromosome Loss and Sensitivity to TBZ, Related to Figure 1

(A) Minichromosome loss assay. The 530kb minichromosome *Ch16* carries the *ade6-216* allele that complements the *ade6-210* allele, resulting in white, *ade6*$^{+}$ expressing colonies. Wild-type or mutant cells containing the *Ch16* minichromosome were grown in media lacking adenine (to select for retention of the plasmid) and then plated on media containing limiting adenine. A minichromosome loss event in the first division following plating results in a colony that is sectored half red/half white. The rate of minichromsome loss per division is therefore indicated by the percentage of half-sectored colonies.

(B) TBZ sensitivity assay. Wild-type or mutant cells were plated on non-selective media (N/S), or media containing the microtubule-destablising drug thiabendazole.

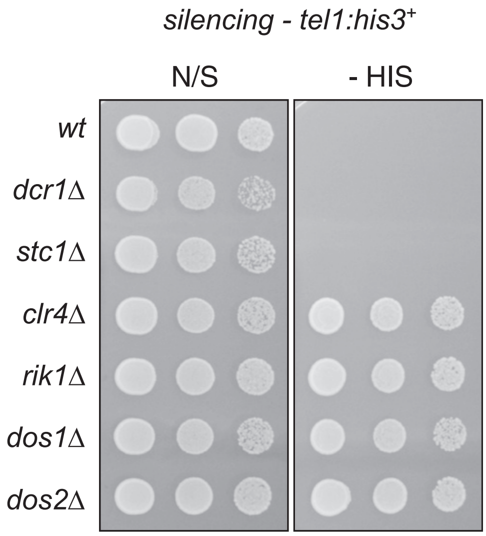

Figure S2. Stc1 Is Not Required for Maintenance of Silencing at Telomeres, Related to Figure 2

Assay for silencing at a telomeric *his3*+ marker gene (*tel1:his3*+) in wild-type or mutant backgrounds. Plates are non-selective (N/S) or lacking histidine (-HIS); growth on -HIS indicates loss of silencing.

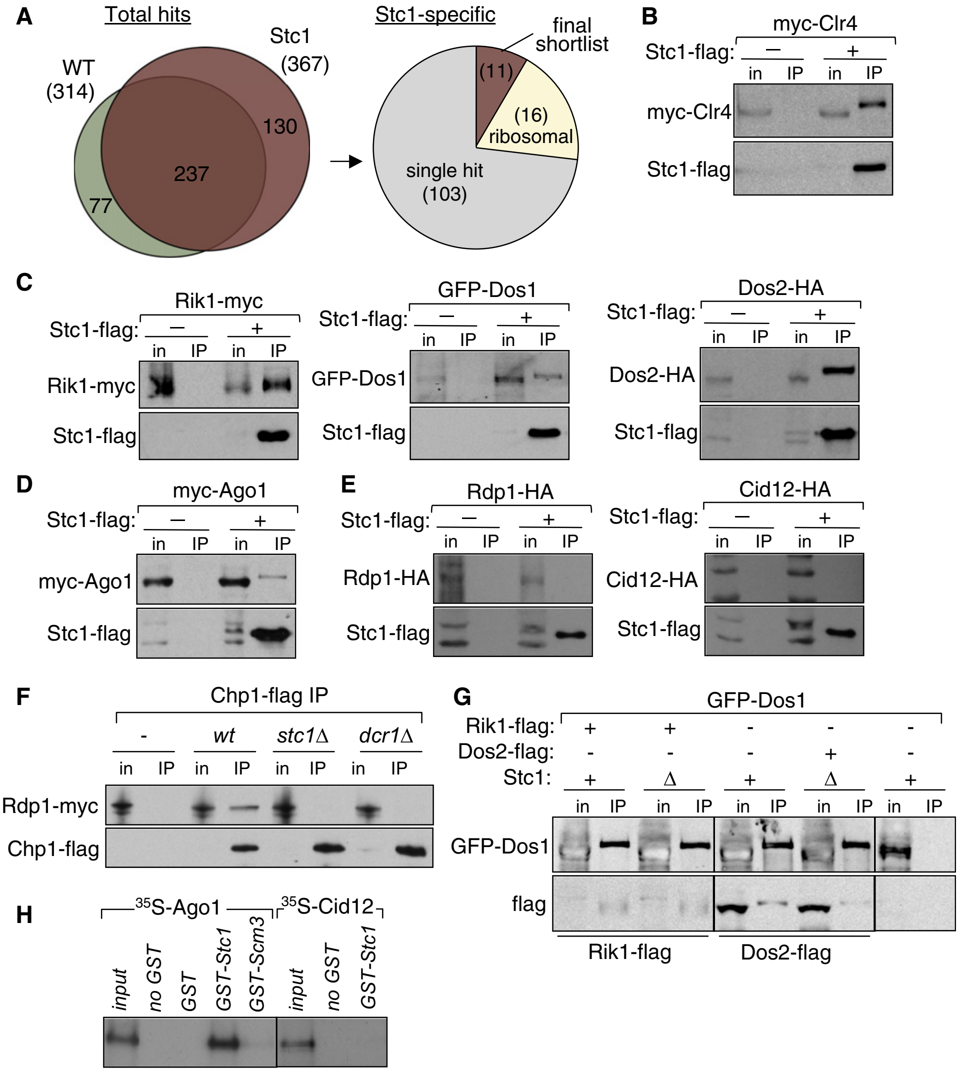

Figure S3. Related to Figure 3

(A) Overview of analysis of proteins associated with affinity purified Stc1-FLAG by mass spectrometry. Four independent FLAG affinity purifications were performed on cells expressing either Stc1-FLAG or untagged Stc1 (WT), and co-precipitating proteins were identified by LC-MS/MS. Complete lists of identified proteins are in Tables S3 and S4. Of 367 proteins identified in Stc1-FLAG immunoprecipitates, 237 were discarded because they were also found in WT. Of the 130 proteins identified specifically in Stc1-FLAG immuno-precipitates, 103 were discarded on the basis that they were identified in less than two of the four replicates (single hit), and a further 16 were discarded because they were ribosomal proteins, which are common contaminants. This left eleven proteins which formed the final shortlist of specific and reproducible Stc1-associated proteins presented in Figure 3.

(B–E) Stc1-FLAG IP followed by western analysis demonstrating that Stc1 associates with CLRC components and Ago1, but not with RDRC components.

(F) Chp1-FLAG IP followed by western analysis, demonstrating that association of Rdp1 with Chp1 depends on Stc1 and Dcr1.

(G) IP of Rik1-FLAG or Dos2-FLAG in wild-type or *stc1Δ* backgrounds, followed by western analysis of associated GFP-Dos1, demonstrating that interactions between CLRC components are unaffected by deletion of Stc1.

(H) In vitro binding assay showing specific interaction of 35S-labeled Ago1 with recombinant GST-Stc1. GST-Scm3 and 35S-Cid12 are negative controls.

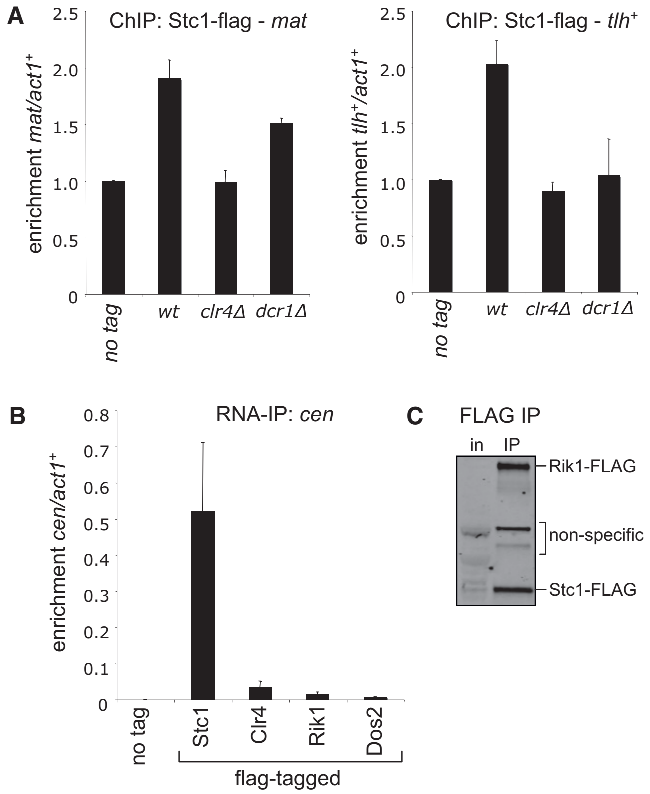

Figure S4. Related to Figure 4

(A) Stc1 associates with the silent mating-type locus and with telomeres. ChIP analysis of Stc1-FLAG association with the silent mating-type locus *(mat)* or the telomere-associated *tlh*$^{+}$genes, relative to *act1*$^{+}$, in wild-type or mutant backgrounds.

(B and C) Stc1 specifically associates with centromere transcripts.

(B) RNA-immunoprecipitation (RNA-IP) analysis of *cen* transcripts associated with FLAG-tagged Stc1, Clr4, Rik1 or Dos2 under native conditions.

(C) Western analysis of FLAG immunoprecipitates from a single strain expressing both FLAG-tagged Rik1 and Stc1, showing that similar amounts of the two proteins are immunoprecipitated (Rik1 1.1x Stc1). Differences in amounts of RNA found in the immunoprecipitates are therefore not due to differences in amounts of protein pulled down.

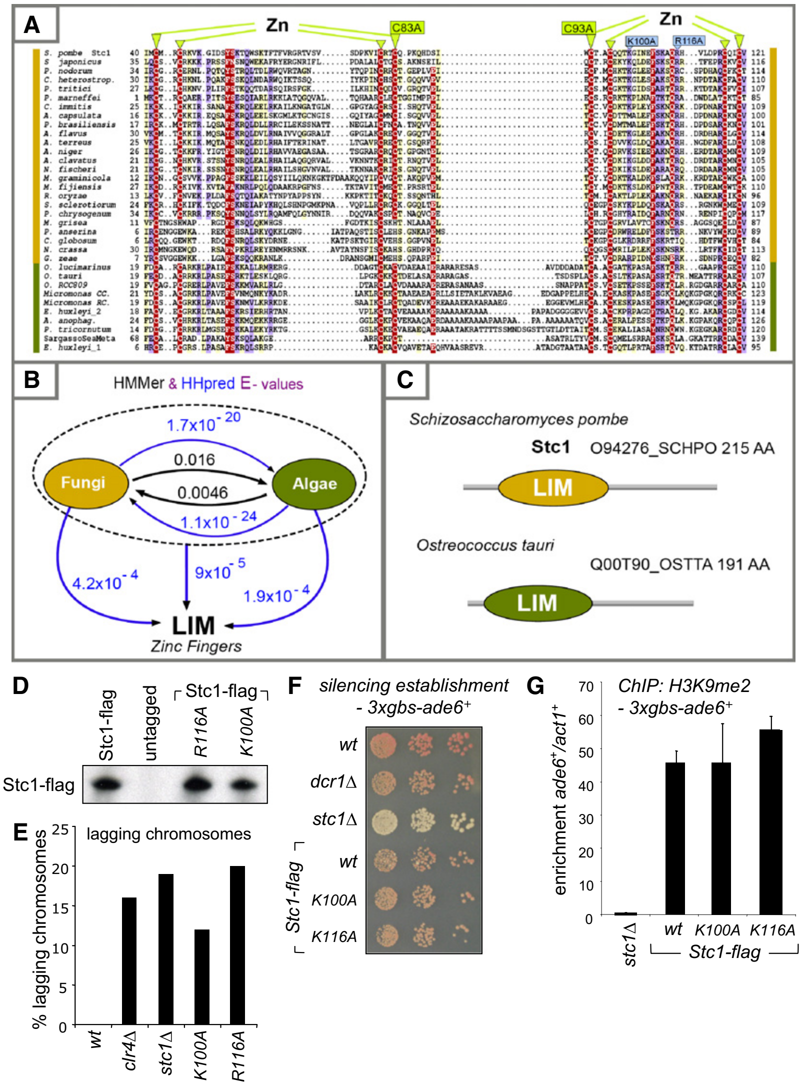

Figure S5. Stc1 Contains a LIM Domain Required for Its Function, Related to Figure 6

(A) Representative multiple sequence alignment of the N-terminal conserved region of Stc1 homologous proteins. Mutated residues mentioned in the text (C83A, C93A, K100A and R116A) are highlighted. The phyletic groups of Stc1 homologous proteins are indicated by colored bars at each side of the alignment: yellow (fungi) and green (algae). The alignment coloring scheme provides an indication of average BLOSUM62 scores (correlated with amino acid conservation) for each alignment column: red (greater than 3), violet (between 3 and 1) and light yellow (between 1 and 0.3). Sequences were obtained from UniProt, GenBank and DOE-JGI (Joint Genome Institute) databases, but were supplemented by manually assembled ESTs and FGENESH$^+$ predicted gene models (Softberry). Sequences are named according to their species abbreviation. Database of origin, accession numbers ("mod" prefix identifies UniProt sequences corrected by gene prediction software FGENESH$^+$) and full species names are: In fungi: O94276_SCHPO Stc1, *Schizosaccharomyces pombe*; B6K2E2_SCHJY, *SchizoSaccharomyces japonicus*; modQ0UKA2_PHANO, *Phaeosphaeria nodorum*; jgi_Coche, *Cochliobolus heterostrophus*; B2WPU1_PYRTR, *Pyrenophora tritici-repentis*; B6QC22_PENMQ, *Penicillium marneffei*; Q1DX71_COCIM, *Coccidioides immitis*; modA6RAF6_AJECN, *Ajellomyces capsulata*; modC1GI81_PARBR, *Paracoccidioides brasiliensis*; B8NGM5_ASPFN, *Aspergillus flavus*; Q0CYE9_ASPTN, *Aspergillus terreus*; modA2QC11_ASPNC, *Aspergillus niger*; modA1C6Z3_ASPCL, *Aspergillus clavatus*; A1DHM0_NEOFI, *Neosartorya fischeri*; jgi_Mycgr, *Mycosphaerella graminicola*; jgi_Mycfi, *Mycosphaerella fijiensis*; est1_Rhior EE004662, *Rhizopus oryzae*; A7EYF8_SCLS1, *Sclerotinia sclerotiorum*; B6GWR5_PENCW, *Penicillium chrysogenum*; modA4RHU1_MAGGR, *Magnaporthe grisea*; modB2B0Z8_PODAN, *Podospora anserina*; Q2GNQ5_CHAGB, *Chaetomium globosum*; Q96U02_NEUCR, *Neurospora crassa*;UPI_GIBZE UPI000023D198, *Gibberella zeae*. In algae: jgi_Ostlu, *Ostreococcus lucimarinus*; Q00T90_OSTTA, *Ostreococcus tauri*; jgi_OstRC, *Ostreococcus RCC809*; jgi_MicCC, *Micromonas CCMP1545*; C1E4D8_9CHLO, *Micromonas sp. RCC299*; jgi2_Emihu, *Emiliania huxleyi*; jgi_Auran, *Aureococcus anophagefferens*; modB7GAR1_PHATR, *Phaeodactylum tricornutum*; SargassoSeaMeta AACY022272749, Sargasso Sea Metagenome; jgi1_Emihu, *Emiliania huxleyi*.

(B) The N terminus of Stc1 bears statistically significant similarity to LIM domains. Numbers correspond to global profile-to-sequence (in black) and profile-to-profile (in blue) comparison E-values obtained from HMMer and HHpred, respectively (Eddy, 1996; Söding et al., 2005). Arrows indicate the profile search direction.

(C) Domain architectures of two members of the Stc1 family.

(D) Stc1^{K100A} and Stc1^{R116A} LIM domain mutant proteins are stable. FLAG-tagged wild-type and mutant Stc1 proteins were affinity purified from cell lysates and

analyzed by western using anti-FLAG antibody.

(E) Stc1 LIM domain mutants exhibit high rates of lagging chromosomes consistent with defects in centromere silencing.

(F) Assay for silencing of *3xgbs-ade6*$^{+}$, mediated by tethered Clr4 (GBD-Clr4-Δcd). The GBD-Clr4-Δcd and 3xgbs-ade6^{+} constructs were combined in either wild-type or mutant backgrounds to assess establishment of silencing upon recruitment of GBD-Clr4. Establishment of silencing at *3xgbs-ade6*$^{+}$ results in red colonies on limiting adenine; if silencing is not established colonies remain white.

(G) ChIP analysis of H3K9me2 levels on *3xgbs-ade6*$^{+}$.

Eddy, S.R. (1996). Hidden Markov models. Curr. Opin. Struct. Biol. *6*, 361–365.

Söding, J., Biegert, A., and Lupas, A.N. (2005). The HHpred interactive server for protein homology detection and structure prediction. Nucleic Acids Res. *33* (*Web Server issue*), W244–W248.
